# Joining of Al 5052 Alloy to Advanced High-Strength Steel Sheets of Dissimilar Thickness Using Laser-Assisted Friction Stir Welding

**DOI:** 10.3390/ma19214677

**Published:** 2026-11-03

**Authors:** Kyounghak Kim, Heeseon Bang

**Affiliations:** 1 Research Institute, Korea Welding and Joining Engineering Association, Jeonnam-Gwangju Special Metropolitan City 61012, Republic of Korea; 2 Department of Welding and Joining Science Engineering, Chosun University, Jeonnam-Gwangju Special Metropolitan City 61452, Republic of Korea

**Keywords:** laser assisted friction stir welding, preheating, dissimilar joint, mechanical characteristics, intermetallic compound, fracture

## Abstract

Multi-material mixed structures (MMMS) are becoming increasingly important to meet the demand for lightweighting in the automotive industry. In this study, the joining of 2.5 mm thick Al 5052 alloy and 1.4 mm thick DP 590 steel sheets was conducted using laser-assisted friction stir welding (LAFSW). A parametric study varying rotational and travel speeds was performed to investigate the effects of process parameters on tensile strength and axial force. The highest tensile strength obtained for the LAFS welds was 193 MPa, which corresponds to approximately 88% of the tensile strength of the Al base metal. The axial force during welding was lower than that of conventional friction stir welding (FSW) due to the thermal softening induced by laser preheating. An intermetallic compound (IMC) layer with a thickness of approximately 5 μm was observed at the joint interface. X-ray diffraction (XRD) analysis of the Fe/Al interface indicated that the IMC layer was composed of FeAl, Fe_2_Al_5_, and Fe_4_Al_13_. Furthermore, minute fragments of IMCs and steel particles dispersed in the nugget zone contributed to the joint strength by acting as reinforcements within the Al matrix. These results suggest that the LAFSW process is an effective alternative to FSW for Al/steel dissimilar joining.

## 1. Introduction

Global regulations on emission reduction and fuel efficiency improvement in the automotive industry have become increasingly stringent in recent years. In response to these growing demands, vehicle lightweighting is playing an essential role, as a 10% reduction in vehicle weight can yield a 6–8% improvement in fuel efficiency. One of the most effective strategies for vehicle lightweighting is multi-material design, which integrates lightweight materials such as aluminum alloys, magnesium alloys, advanced high-strength steels (AHSS), and carbon-fiber-reinforced plastics (CFRP) to achieve substantial weight reduction. Consequently, multi-material structures combining aluminum alloys with advanced high-strength steels are increasingly required to improve the strength-to-weight ratio of structural components.

However, joining aluminum alloys to steel remains challenging in structural manufacturing. The primary obstacles causing joint failure between these dissimilar materials are the significant discrepancies in their chemical and thermophysical properties (e.g., melting point, thermal conductivity, and coefficient of thermal expansion) along with the formation of brittle intermetallic compounds (IMCs). The growth of a brittle IMC layer at the Al/steel interface severely degrades the mechanical properties of the joint. Because an excessively thick IMC layer induces joint embrittlement and promotes crack initiation and propagation, the layer thickness should ideally be restricted to less than 10 μm [1,2]. Therefore, suppressing excessive formation of intermetallic compounds is vital for the dissimilar joining of aluminum to steel. In this regard, the successful integration of aluminum alloys and steel offers substantial flexibility in the design and functionality of engineering structures. Furthermore, a comprehensive evaluation of joint integrity encompassing mechanical properties, microstructural characteristics, and corrosion behavior must be rigorously conducted for automotive applications.

Dissimilar Al/steel joints can be produced via thermal joining (involving solid/solid, solid/liquid, or liquid/liquid interfaces), mechanical fastening (e.g., clinching, riveting, and bolting), and adhesive bonding [3]. In thermal joining, solid/solid interface processes operate below the melting point of the aluminum alloy; these include diffusion bonding [4], magnetic pulse welding [5], ultrasonic welding [6], friction stir welding (FSW) [7], and hybrid friction stir welding [8]. In solid/liquid interface joining, such as arc brazing [9], laser brazing [10], and resistance spot welding [11], the peak temperature exceeds the melting point of the aluminum alloy but remains below that of the steel, allowing the molten aluminum to wet the solid steel substrate. In liquid/liquid interface joining, where the peak temperature exceeds the melting point of steel, laser beam welding is predominantly employed [12]. While adhesive bonding and mechanical fastening are also widely adopted in the automotive sector, both present notable drawbacks. Adhesive bonding requires extended curing times and raises environmental concerns, whereas mechanical fastening inevitably increases component weight and induces severe stress concentrations around fastener locations [13].

FSW, as a solid-state joining technology, has emerged as a prominent method for joining both similar and dissimilar materials. Compared with conventional fusion welding, this solid-state process operates at lower heat inputs, thereby mitigating solidification defects such as hot cracking and porosity [14]. Additionally, FSW delivers superior as-welded mechanical properties, minimal distortion, and reduced residual stresses. Crucially, it suppresses Fe–Al intermetallic compound growth because solid-state interdiffusion between Fe and Al is significantly slower than in the liquid phase. Furthermore, hybrid joining processes that combine FSW with auxiliary energy sources have emerged as viable alternatives for dissimilar material combinations. For instance, synergistic coupling between FSW and a secondary heat source positively influences heat generation, material flow, and overall joint integrity.

Several studies have investigated gas tungsten arc welding assisted friction stir welding (GAFSW) and laser assisted friction stir welding (LAFSW) for Al/steel joining. GAFSW was applied to join 3 mm thick 304L stainless steel to 3 mm thick Al 6061-T6, achieving a maximum tensile strength 15% higher than that of FSW [8]. The authors attributed this enhancement to improved plastic material flow and localized annealing resulting from GTAW preheating on the harder steel side. This beneficial preheating effect on joint performance was corroborated by subsequent studies [15,16,17]. Regarding laser-assisted methods, diode and Nd:YAG lasers are the primary sources used to preheat the harder material locally. LAFSW using a diode laser was utilized to join 1 mm thick DC04 steel to 1 mm thick Al 6016-T4, achieving approximately 80% of the tensile strength of the Al base metal while maintaining excellent formability in drawable steel–aluminum tailored blanks [18]. Similarly, LAFSW with an Nd:YAG laser demonstrated superior tensile strength compared to FSW due to enhanced material flow promoted by laser preheating [19,20]. Although dissimilar Al/steel joining has been extensively explored, studies on LAFSW applied to Al/steel joints with dissimilar sheet thicknesses remain scarce.

**Figure 1:**
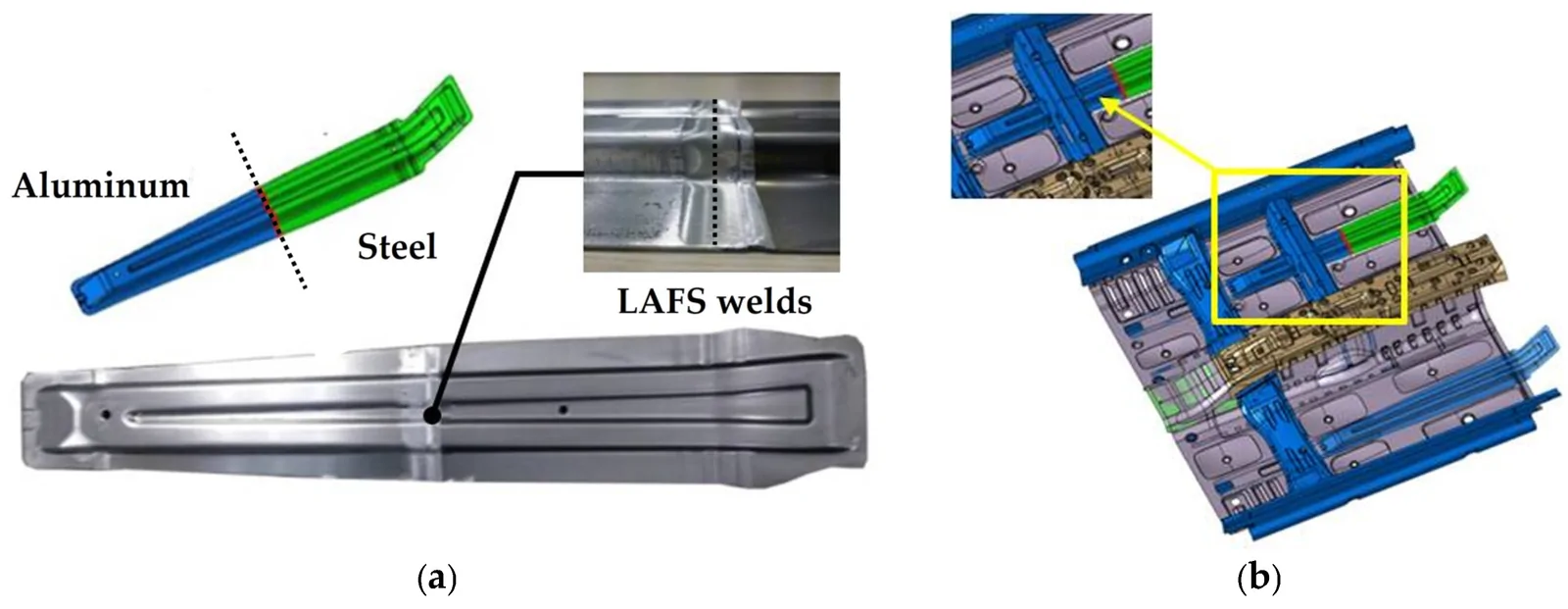
Configuration of the automotive components. (**a**) Side upper; (**b**) center floor [21].

Therefore, the present study evaluates the feasibility of LAFSW for automotive manufacturing by joining sheets of dissimilar materials and thicknesses: a 2.5 mm thick Al alloy and a 1.4 mm thick steel. The effects of key process parameters on the metallurgical and mechanical characteristics of the joints were systematically investigated. Furthermore, as shown in Figure 1a, this advanced LAFSW process was applied to fabricate an automotive side upper component to achieve vehicle weight reduction. Figure 1b illustrates the final lightweight center-floor module assembled with the LAFSW-processed side-upper component.

## 2. Materials and Methods

### 2.1. Experimental Set-Up

**Figure 2:**
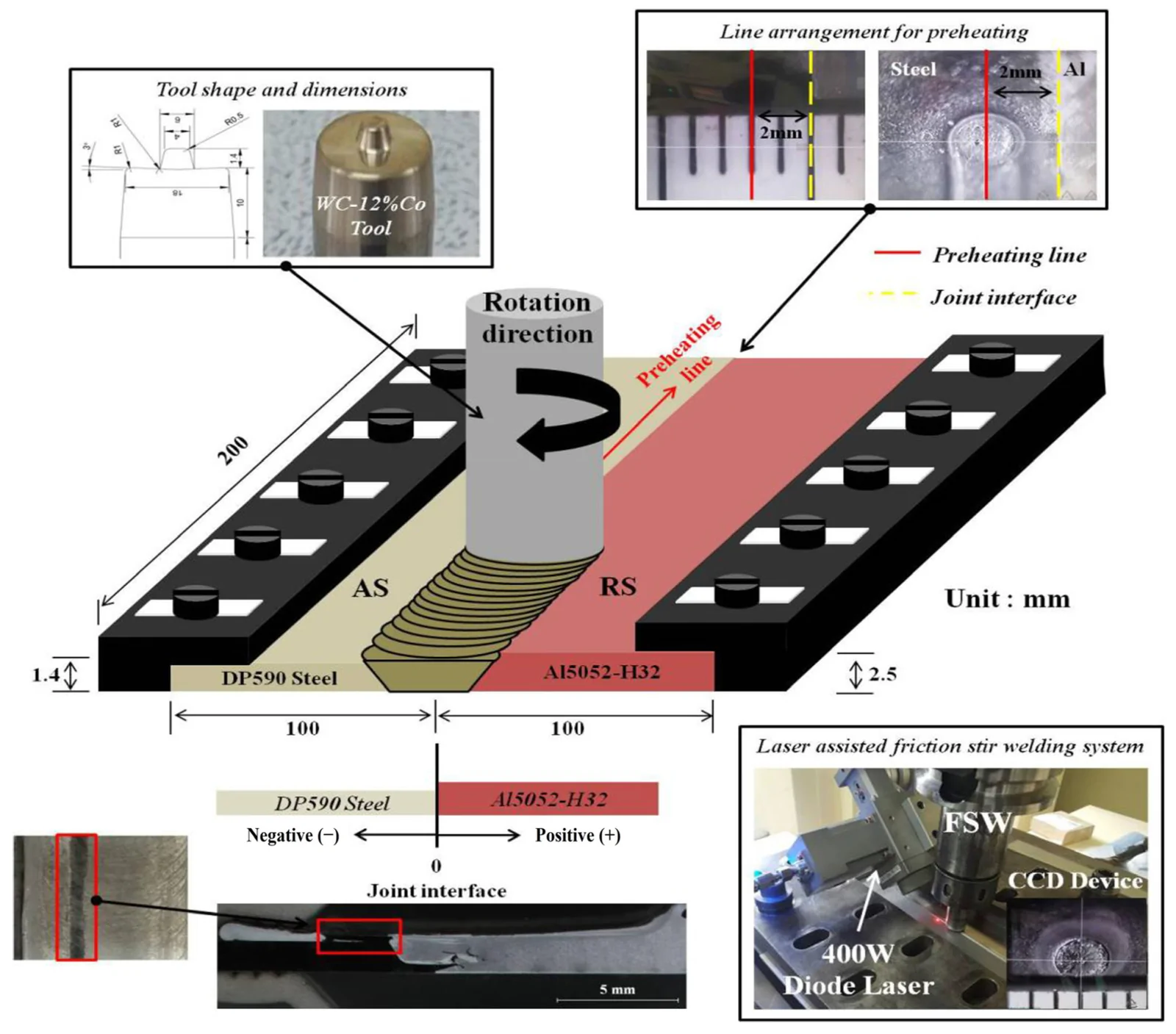
Schematic illustration for LFSW system [21].

In the present study, a 2.5 mm thick AA5052-H32 and a 1.4 mm thick SPFC590DP steel sheet were used to fabricate dissimilar-thickness tailored blanks via LAFSW. The experimental set-up with a joint configuration of 200 mm (L) × 200 mm (W) is schematically illustrated in Figure 2. AA5052-H32, a non-heat-treatable Al–Mg alloy exhibiting favorable strength and corrosion resistance, is widely utilized for structural components in the automotive industry. SPFC590DP steel, a cold-rolled dual-phase (DP) steel composed of a martensitic phase within a ferritic matrix, has been broadly adopted as an AHSS for vehicle lightweighting. The chemical compositions and mechanical properties of the base materials are summarized in Table 1. Prior to welding, the faying surfaces of all base plates were degreased and cleaned with acetone to eliminate surface contaminants. The AA5052-H32 and SPFC590DP sheets were positioned on the retreating side (RS) and advancing side (AS), respectively. As reported by Watanabe et al. [7], positioning the harder material on the AS and applying a pin offset toward the steel faying surface are essential for sound joint consolidation. A tapered cylindrical FSW tool fabricated from tungsten carbide with 12 wt.% cobalt (WC–12%Co, hardness of 1522 HV; KONASOL, Inc., Dangjin-si, Korea) was employed. The tool geometry featured a concave shoulder and a conical frustum pin. The specific tool dimensions were as follows: shoulder diameter of 18 mm, pin top and bottom diameters of 6 mm and 4 mm, respectively, and a pin length of 1.4 mm. A tool tilt angle of 3° was maintained throughout the welding process. Table 2 lists the welding parameters investigated in this work. The LAFSW system, integrated with a charge-coupled device (CCD) camera, was developed for dissimilar joining. An industrial 400 W continuous wave diode laser (UW-400-915, United Winner Laser Ltd., Shenzhen, China) was utilized to locally preheat the steel sheet. The CCD camera was implemented to ensure precise real-time alignment of the pin offset and the laser preheating trajectory. All LAFSW trials were performed in displacement control mode. A load cell was utilized to examine the effect of process parameters on the downward axial force (Fz) during welding. The leading laser beam irradiated a target spot on the steel sheet located 2 mm away from the joint interface at an incident angle of 45°, with a fixed distance of 5 mm between the tool axis and the laser spot center. This preheating strategy was implemented to mitigate tool wear and facilitate plastic material flow during welding.

### 2.2. Mechanical Test

**Figure 3:**
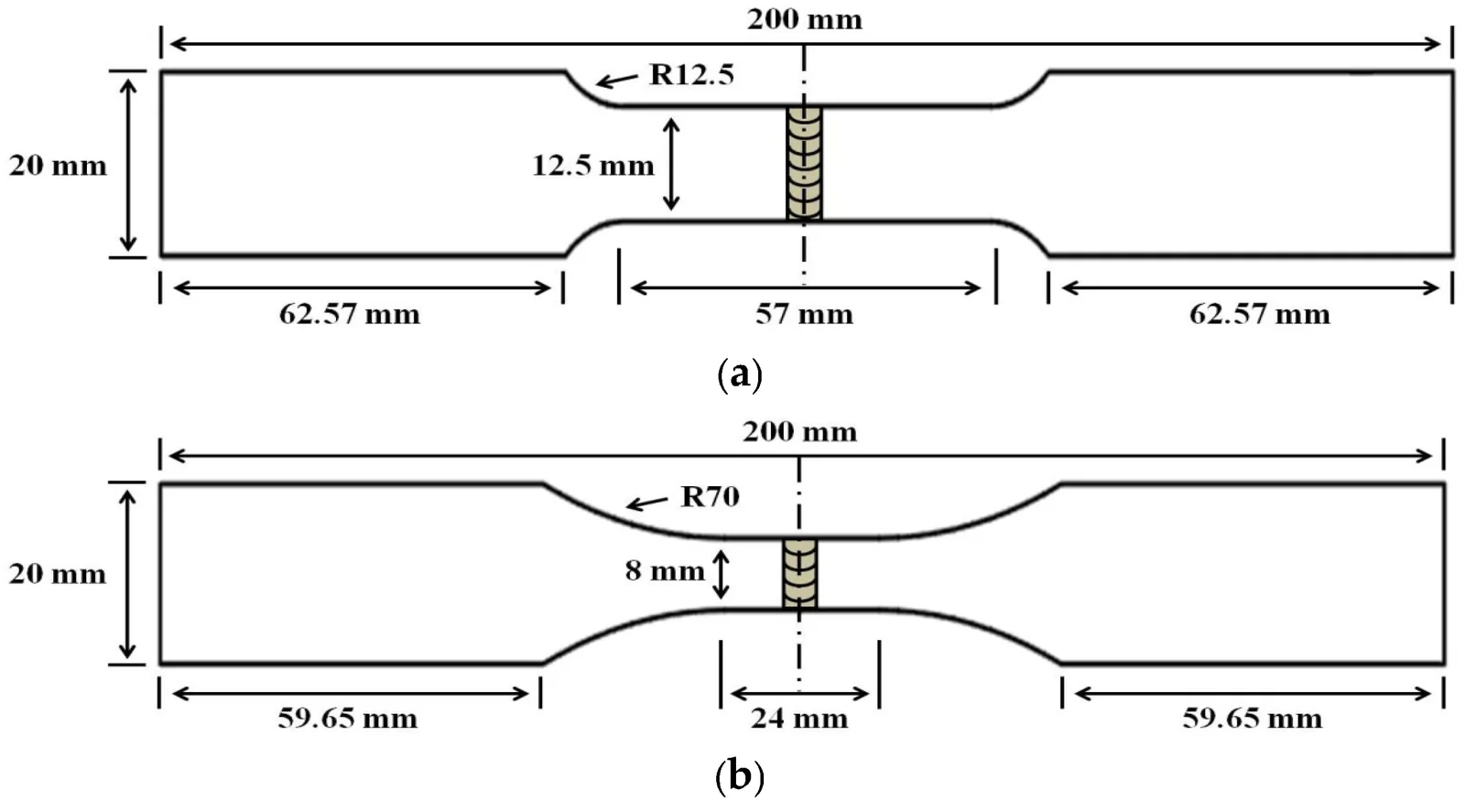
Dimensions of standard specimens: (**a**) Tensile specimen, and (**b**) Fatigue specimen [21].

Figure 3 shows the geometries and dimensions of the standard tensile and fatigue specimens. Both tensile and fatigue specimens were extracted perpendicular to the welding direction using a wire electrical discharge machining system, with the tensile load axis aligned perpendicular to the welds. Tensile tests were performed at room temperature using a universal testing machine (EHF-EF200kN, Shimadzu, Kyoto-shi, Japan) at a crosshead speed of 1 mm/min in accordance with the ASTM E8 standard. Prior to fatigue testing, all fatigue specimens were manually polished. Uniaxial fatigue tests were conducted at room temperature in accordance with the ASTM E466 standard using a servo-hydraulic fatigue testing machine (661.20F-02, 50 kN load capacity, MTS Systems Corporation, Eden Prairie, MN, USA). The cyclic loading was applied in a tension–tension mode with a sinusoidal waveform at a frequency of 10 Hz and a stress ratio of R = 0.1. The initial maximum cyclic stress level for the fatigue test was set to 60% of the ultimate tensile strength (0.6σT). To ensure reproducibility, both tensile and fatigue tests were performed three times for each condition. Furthermore, the micro-Vickers hardness (AKASHI HM112, Mitutoyo, Kawasaki-shi, Japan) was measured across the mid-thickness of the transverse joint cross-section under an indentation load of 500 g with a dwell time of 10 s.

### 2.3. Metallurgical Test

All samples for metallurgical observation were polished from emery papers of 400 to 4000 grit and diamond suspension of 9, 3 and 1 micron. After polishing, the metallographic samples were etched in 5% Nital reagent (100 mL Ethanol + 5 mL HNO_3_) for 5 s and Tucker’s reagent (45 mL HCl + 15 mL HNO_3_ + 15 mL HF + 25 mL distilled water) for 10 s in sequence to observe the macrostructure using an optical microscope (SZ61 and BX51M with I solution imaging program, Olympus, Tokyo-shi, Japan). The thickness and composition of the interfacial layer between Al and Fe were characterized by scanning electron microscopy (SEM) with energy-dispersive spectrometry (EDS) using a beam energy of 15 kV and magnification of ×5000. In addition, the phase of the intermetallic compound formed at the joint interface was identified by X-ray diffractometer (XRD) using monochromatic CuKa radiation. The fracture surface of the tensile specimen was characterized using SEM.

## 3. Results and Discussion

### 3.1. Bead Profile and Cross Section of LAFS Welds

Table 3 shows bead profiles of LAFS welds produced at various travel speeds and tool rotational speeds ranging from 300 to 500 rpm, with a constant plunge depth of 0.8 mm, pin offset of 0.9 mm, and laser power of 200 W. The weld bead quality associated with surface material flow improved with decreasing travel speed. When the travel speed increased from 60 to 120 mm/min across all rotational speeds, the bead width significantly decreased, which is largely attributed to the variation in heat input. Furthermore, Table 3 reveals no obvious surface defects, such as surface grooves (or tunnels) and voids, which typically arise from insufficient heat generation and inadequate material intermixing within the nugget zone (NZ) under inappropriate process parameters. An elongated steel strip formed on the AS was observed in all dissimilar joints due to the thickness discrepancy between the Al and the steel. As depicted in Table 3, increasing the travel speed from 60 to 120 mm/min noticeably reduced the width of this elongated steel strip at all rotational speeds, whereas a higher rotational speed led to a wider strip. This behavior can be explained in terms of frictional heat generation between the tool shoulder and the workpiece, primarily governed by travel and rotational speeds.

Table 4 shows the cross-sectional macrostructures of the LAFS welds. No internal defects, such as tunnels or cracks, were detected at the joint interface or within the NZ. Steel fragments dispersed into the Al matrix were non-uniformly distributed within the nugget zone through mechanical stirring. In the AS, the joint interface between the aluminum alloy and steel tilted at a pronounced inclination angle that exceeded the original taper angle of the tool pin. The inclination angle is defined as the angle between the vertical direction and the direction of the joint interface. This inclination angle decreased with increasing travel speed (from 60 to 120 mm/min), and a higher rotational speed similarly led to a smaller angle. Moreover, the curved interface transitioned into a concave geometry as the rotational speed increased from 300 to 500 rpm at a constant travel speed of 60 mm/min.

### 3.2. Mechanical Characteristics

**Figure 4:**
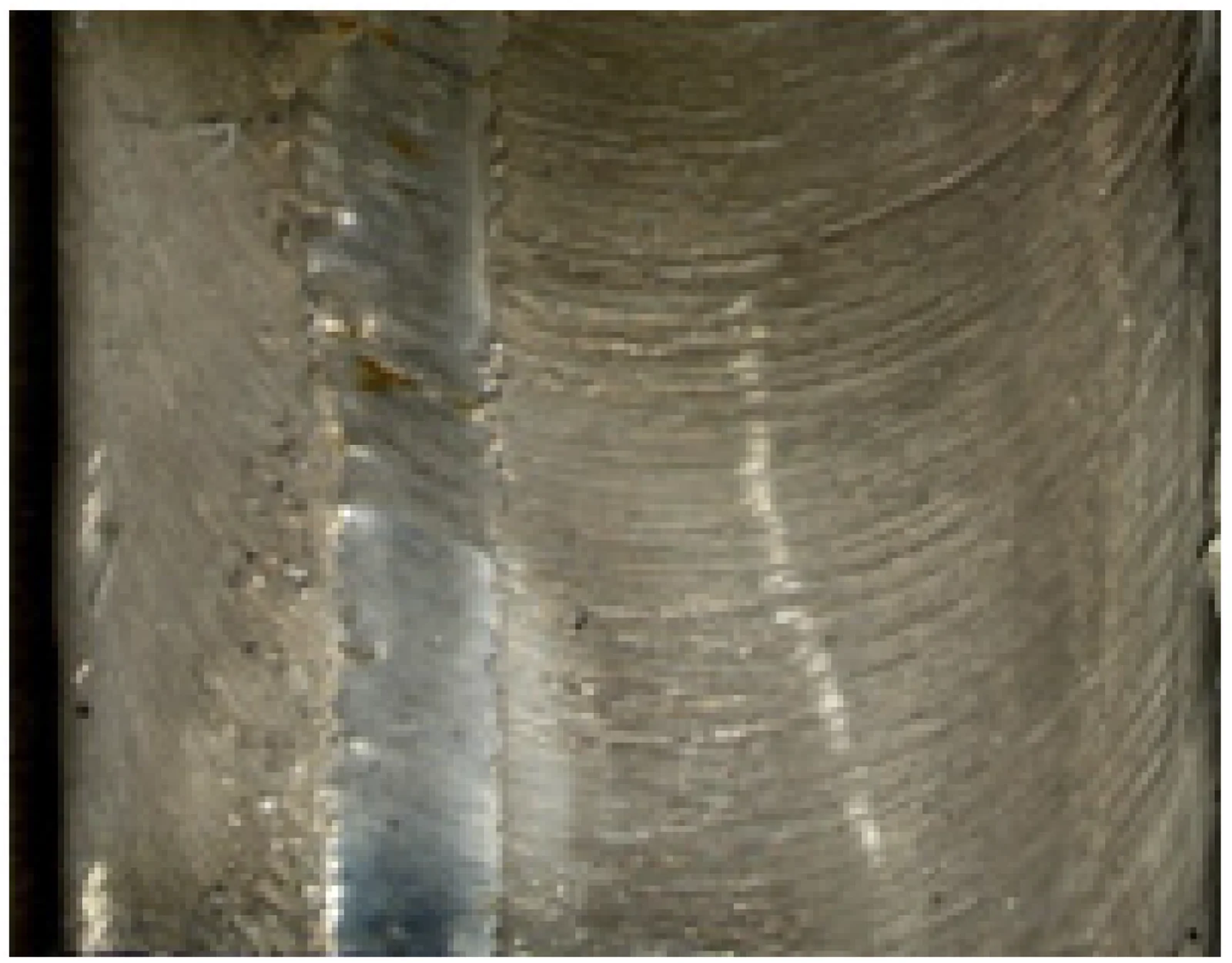
The comparison of tensile strength of LAFS welds with different rotational speeds and travel speeds: (**a**) 300 rpm, (**b**) 400 rpm, and (**c**) 500 rpm [21].

Figure 4 shows the comparison of the tensile strength of LAFS welds fabricated under various process parameters: a travel speed of 60–120 mm/min, a rotational speed of 300–500 rpm, and a laser power of 200 W. The highest tensile strength of the LAFS welds reached 193 MPa, which was obtained at a travel speed of 60 mm/min and a rotational speed of 400 rpm, corresponding to a joint efficiency of approximately 88% of the Al base metal. For the LAFS welds produced at 300 and 500 rpm, the tensile strength increased as the travel speed increased from 60 to 90 mm/min, followed by a decrease at 120 mm/min (Figure 4a,c). In contrast, the tensile strength of the welds produced at 400 rpm monotonically decreased with increasing travel speed from 60 to 120 mm/min (Figure 4b). This behavior differs noticeably from the tensile strength trend observed in FS welds [20]. While the FS welds exhibited their highest tensile strength at the lowest travel speed of 60 mm/min irrespective of rotational speed, the LAFS welds achieved higher tensile strength at faster speeds and demonstrated overall superior strength compared to the FS welds, indicating a joint efficiency of approximately 79%. Therefore, it can be inferred that the improved tensile strength was attributed to sufficient material flow promoted by laser preheating.

**Figure 5:**
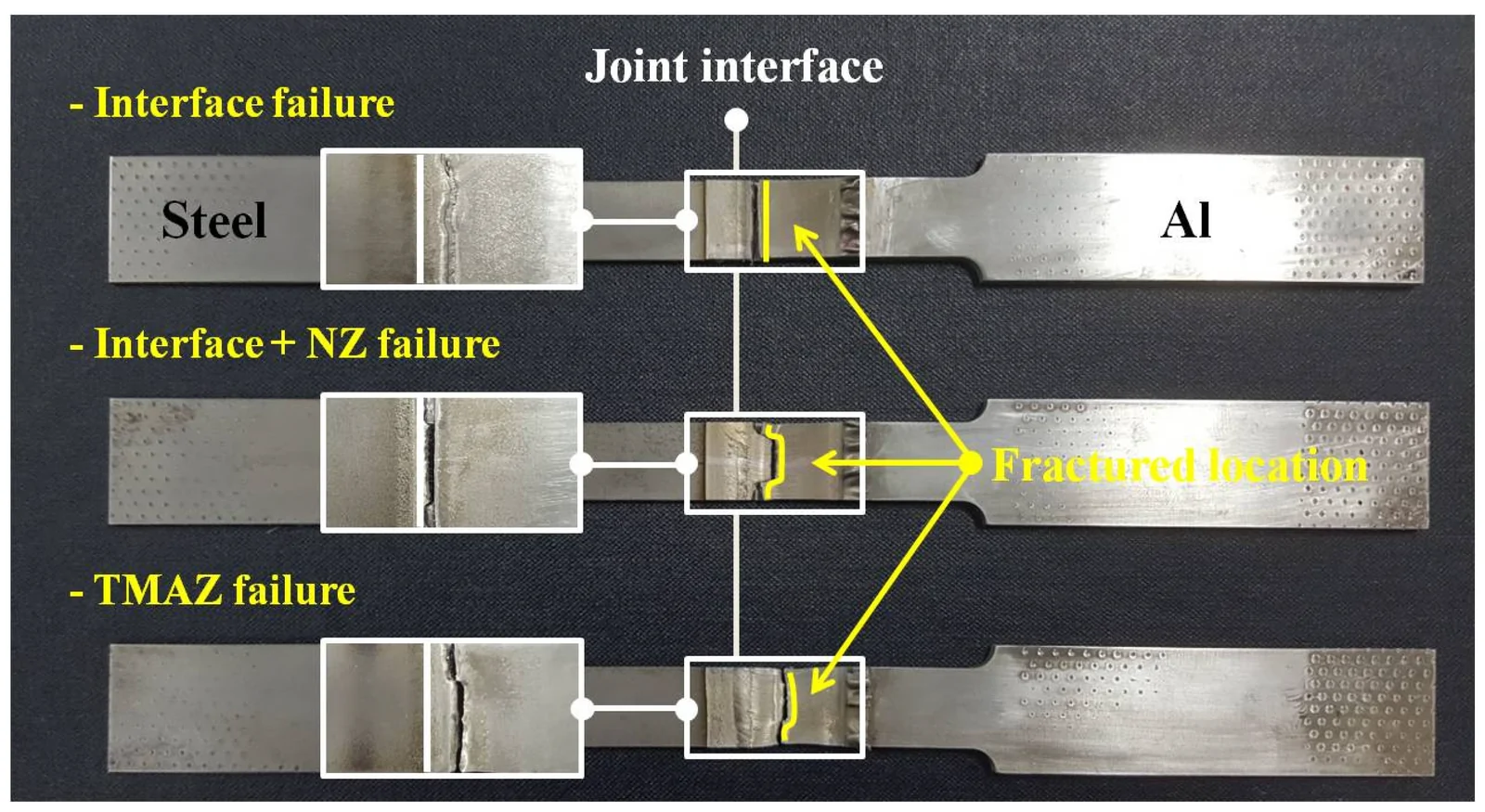
Macrograph of fractured tensile specimen of LAFS welds [21].

Figure 5 presents macrographs of the fractured tensile specimens after testing. Three distinct failure modes were identified: interface failure (predominantly observed at 60 and 90 mm/min), interface + NZ failure (mainly observed at 120 mm/min), and TMAZ (thermo-mechanically affected zone) failure (observed at 60 mm/min and 400 rpm). Interface failure was the most frequent mode in this study. In this failure mode, cracks initiated at the upper region of the LAFS weld and propagated along the joint interface; hence, the fracture behavior of these dissimilar welds was significantly governed by the formation of IMCs. The interface + NZ failure mode, which predominantly occurred at the highest travel speed, yielded the lowest tensile strength among the three modes. This degradation was attributed to the presence of large steel fragments and an unwelded region at the bottom of the joint, which is consistent with the macrostructural observations. As shown in Figure 4, the TMAZ failure mode proved to be the most favorable, resulting in an ultimate tensile strength of 193 MPa (~88% of the Al base metal). The main findings are summarized as follows:

- At welding speeds of 60 and 90 mm/min: The relatively higher heat input results in successful bonding extending down to the bottom of the steel region, and as the tensile load increases, the crack propagates along the interface (interface failure).
- At a welding speed of 120 mm/min (the highest welding speed): An unwelded zone exists in the lower region of the steel, acting as the initiation site for fracture. As the tensile load increases, the crack propagates along the interface or through the steel fragments, eventually leading to failure (Interface + NZ failure).
- At 400 rpm and 60 mm/min (the optimal condition): As the tensile load increases, initial cracks are generated from the IMC particles distributed within the NZ, ultimately leading to fracture in the TMAZ.

**Figure 6:**
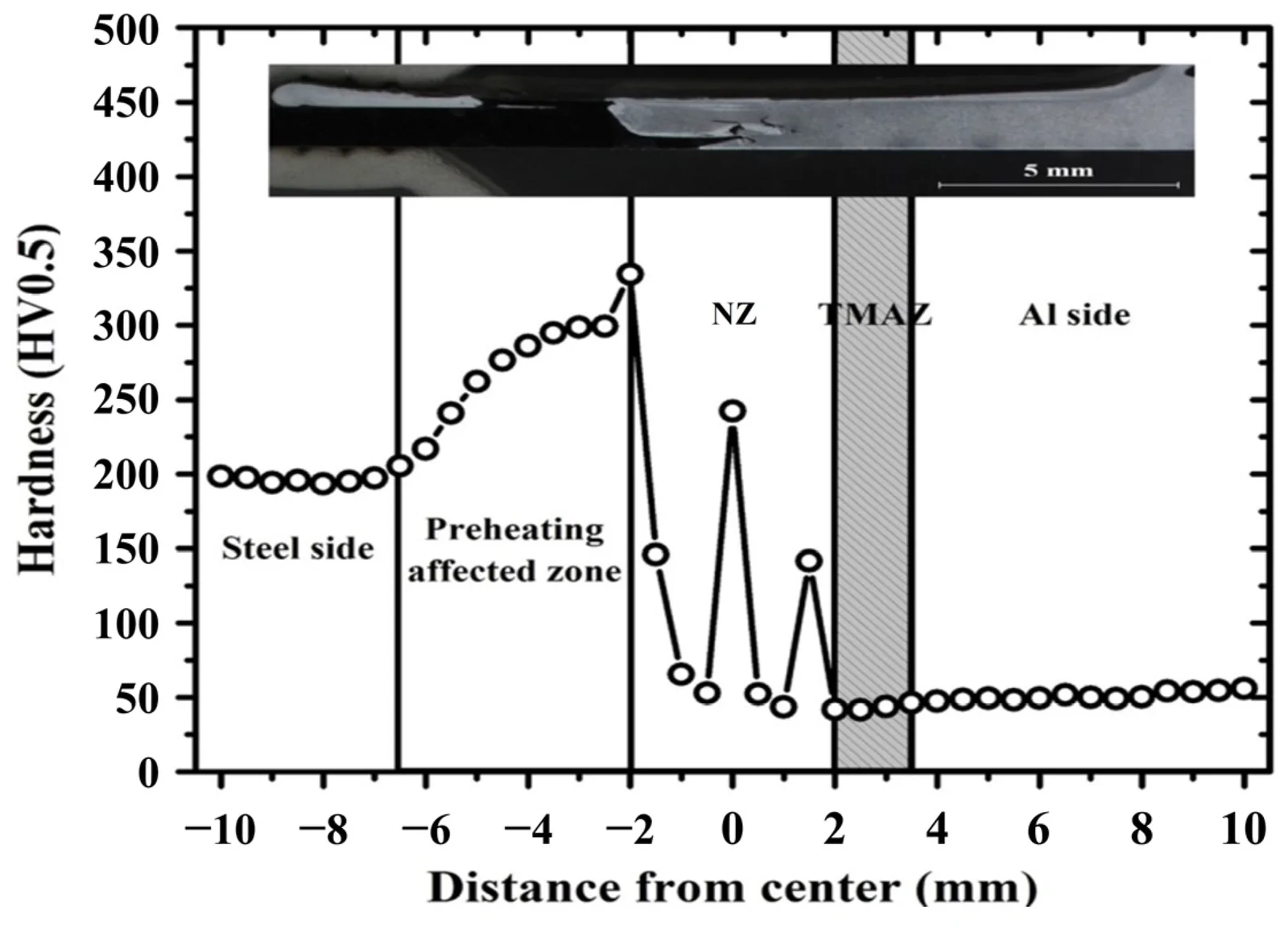
Hardness distribution of LAFS welds along the transverse middle line [21].

Figure 6 shows the hardness distribution measured along the midline of the transverse cross-section of the LAFS welds, which exhibited the highest tensile strength after the tensile test. The base metal hardness values of the Al5052-H32 and DP590 steel used in the present study ranged from 54 to 57 HV and 192 to 198 HV, respectively. Four distinct zones, namely the NZ, TMAZ, preheating-affected zone (PAZ), and base metal (BM), were identified in the hardness distribution across the LAFS welds on both the steel and Al sides. The hardness value on the steel side close to the joint interface was significantly higher than that of the steel base metal due to grain refinement and high hardenability. It also increased in the PAZ on the advancing side due to microstructural variations, specifically a decrease in the ferrite matrix and an increase in martensite, caused by the quenching effect after welding. This decrease in hardness from the joint interface to the steel base metal is in good agreement with the results reported by You et al. [22]. They explained that since the peak temperature in the TMAZ lies between Ac_1_ and Ac_3_, the initial microstructure undergoes partial austenitization during heating, leading to the formation of fine ferrite grains and martensite upon subsequent cooling. In contrast, the peak temperature in the PAZ remains slightly below Ac_1_, resulting in the formation of tempered martensite. The minimum hardness value in the NZ on the RS was about 48 HV, which was lower than that of the BM. A W-shaped pattern was observed in the NZ due to an increase in hardness caused by scattered steel particles. A localized decrease in hardness due to softening was observed in the TMAZ close to the NZ, approximately 4 mm away from the joint interface.

**Figure 7:**
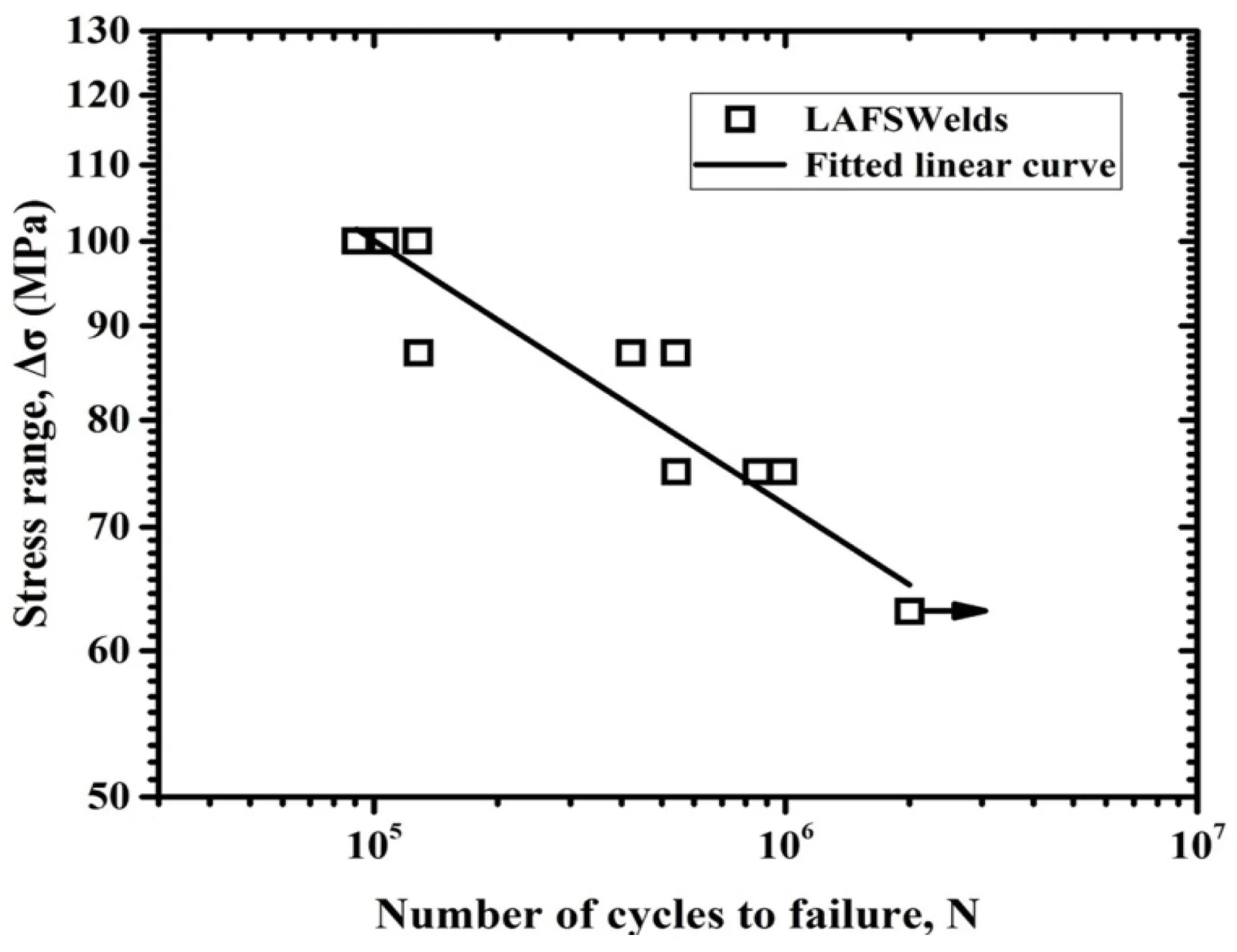
Stress-Number of cycles to failure curve (S-N curve) of LAFS welds produced under welding parameters; at a rotational speed of 400 rpm and at a travel speed of 60 mm/min [21].

Figure 7 shows the stress versus number of cycles to failure (S-N) curve of the LAFS welds, which exhibited the highest tensile strength after the tensile test. Table 5 lists the detailed results of the fatigue tests. To ensure the statistical reliability of the mechanical test results and evaluate measurement repeatability, a total of 12 specimens were tested. Specifically, the fatigue tests were conducted at four distinct stress ranges (Δσ = 100, 87, 75, and 63 MPa), with three replicate specimens tested at each stress level. Based on the fatigue test results, the fatigue life and its statistical scatter exhibited a strong dependency on the applied stress range. At the highest stress level of 100 MPa, the fatigue life was fairly short with a relatively narrow scatter band (108,472 ± 18,930 cycles, CV: 17.5%). In contrast, at the intermediate stress range of 87 MPa, the fatigue life tended to increase but became significantly more scattered (401,541 ± 243,311 cycles, CV: 60.6%). This pronounced variance represents the inherent stochastic nature of fatigue behavior under intermediate cyclic loading. In this regime, the fatigue life becomes highly sensitive to natural statistical variations in crack initiation, resulting in a significantly wider scatter band compared to high-stress conditions. As the stress range decreased further to 75 MPa, the data showed a stable increase in fatigue life along with reduced scatter (891,132 ± 78,893 cycles, CV: 8.9%). Ultimately, at 63 MPa, the LAFS welds yielded the most stable resistance to fatigue failure, with all replicate specimens reaching the run-out limit (2,000,000 ± 0 cycles, CV: 0.0%), statistically confirming a reliable fatigue limit. The fatigue data can be expressed as the stress range (Δσ) versus the corresponding number of cycles to failure (N). A stress level corresponding to 60% of the tensile strength was determined as the first-stage stress level for the fatigue tests. To mathematically account for the statistical variability and variance observed in the replicate tests, a linear regression line was determined using the least-squares method on a logarithmic scale to fit the finite life regime. The fatigue strength of the LAFS welds was established at 63 MPa, which aligns with the run-out condition on the S-N curve at 2 × 10^6^ cycles. The mathematical relationship between the stress level and fatigue life for the welded joint is expressed as follows:

(1) $${\left(\Delta \sigma \right)}^{m}\cdot N=C$$

where Δσ is the stress range, N is the number of cycles to failure, and m and C are the fitting constants related to the material properties in the S-N curve.

### 3.3. Axial Force Characteristics During Welding

**Figure 8:**
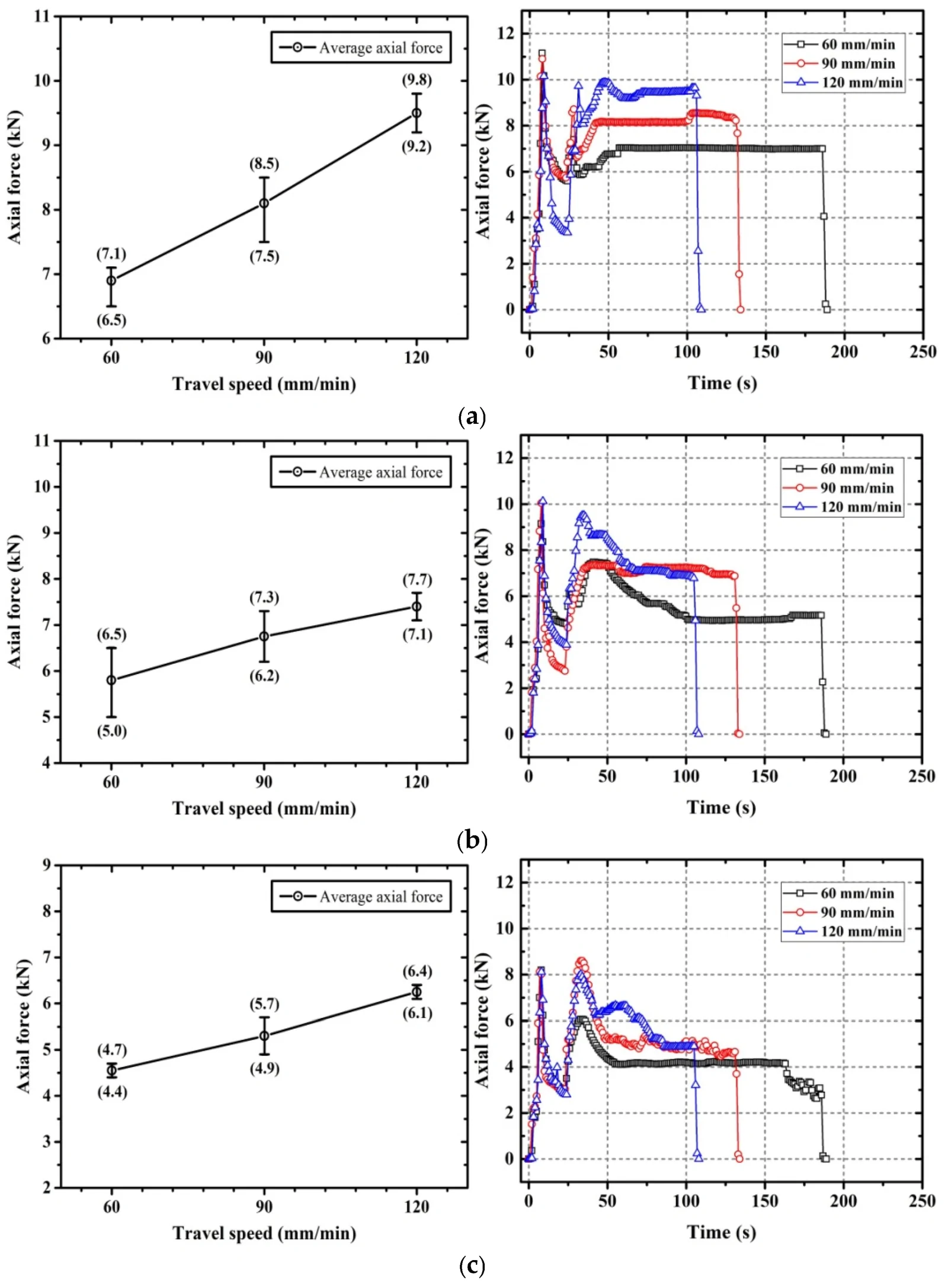
Effect of process parameters on the axial force during welding: (**a**) 300 rpm, (**b**)400 rpm, and (**c**) 500 rpm (left: average axial force, and right: axial force versus time curve) [21].

Figure 8 shows the effect of process parameters on the axial force (F_Z_) during the welding stage under different values of travel speed and rotational speed. Note that the axial force during welding can be determined by monitoring the Z-axis output of the motor. Based on the axial force versus time curve, there are four distinct features indicating the plunging, dwelling, welding, and pulling stages. In the plunging stage, the axial force increases significantly at the beginning. A higher rotational speed, which can increase the material shear strain rate between the pin surface and the material, can reduce the axial force in the plunging stage. Hence, a higher rotational speed contributes to enhancing both plastic material flow and frictional heat generation. It is seen from Figure 8 that a positive relationship is evident between the axial force and the travel speed, as well as between the axial force and the rotational speed. The overall axial force decreases with a decrease in travel speed.

### 3.4. Metallurgical Characteristics

**Figure 9:**
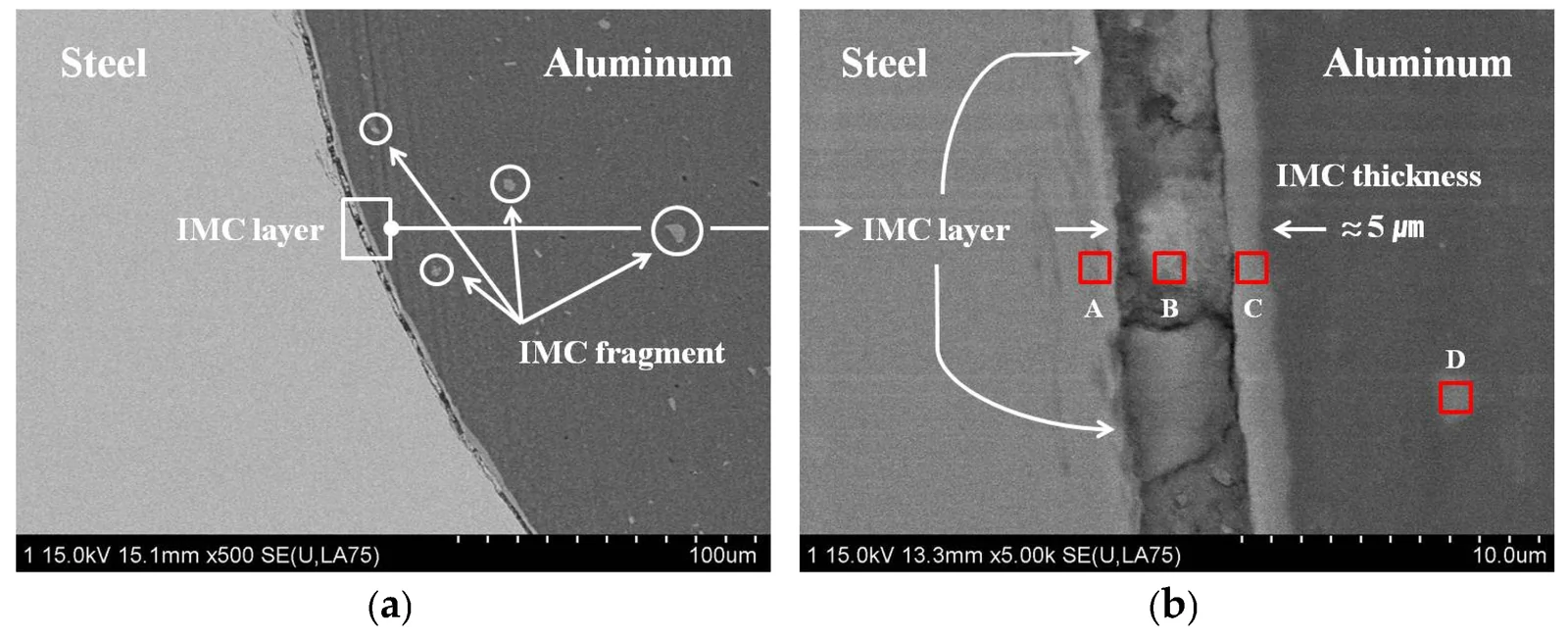
SEM BSE images of microstructure near joint interface; (**a**) Microstructure near joint interface and (**b**) Magnification of (**a**) [21].

**Figure 10:**
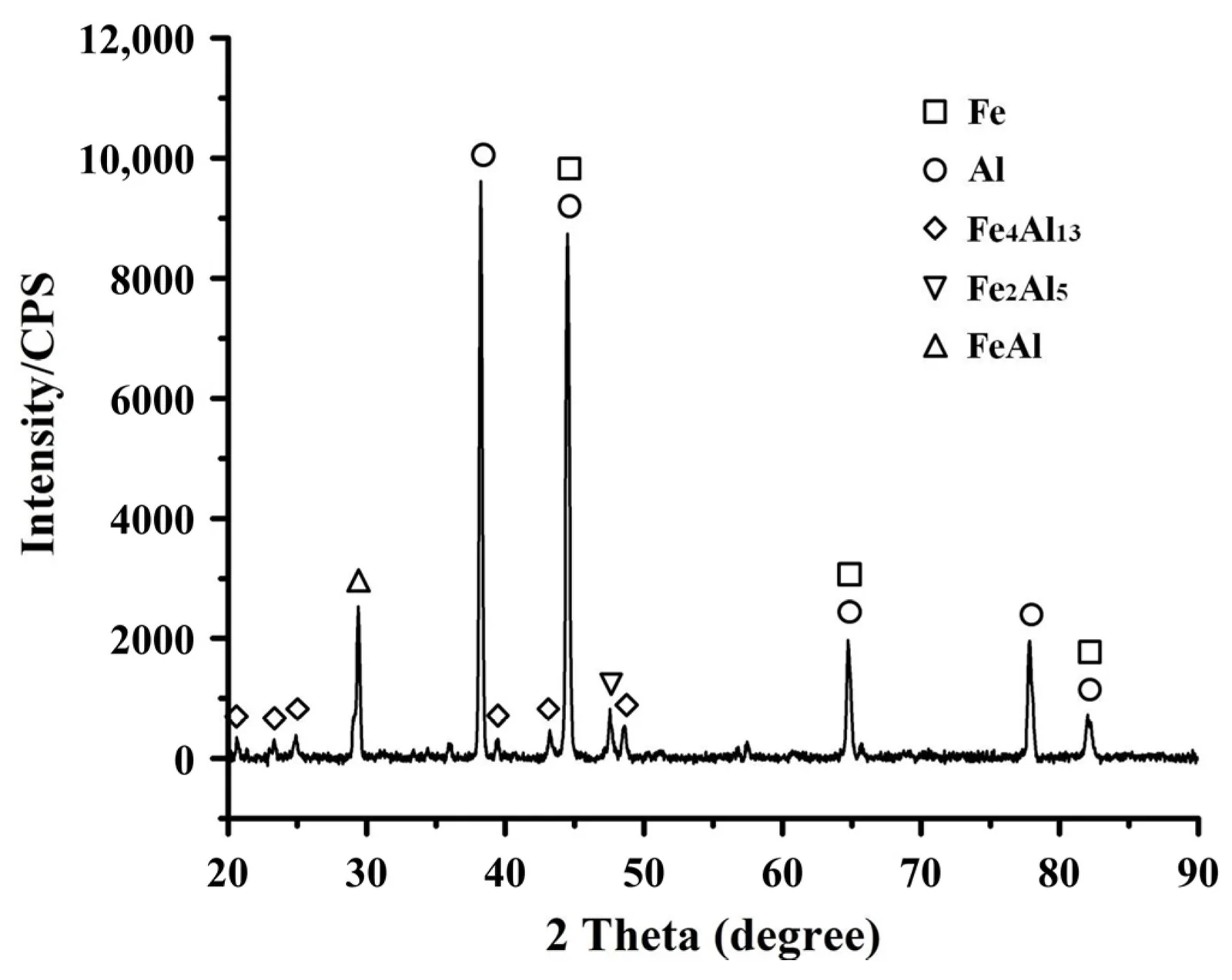
XRD spectrum of LAFS welds [21].

Figure 9 shows the SEM BSE images of the LAFS welds fabricated at a rotational speed of 400 rpm and a travel speed of 60 mm/min, where the observed position was the central region of the IMC layer between Fe (bright side) and Al (dark side). The results obtained from the composition analysis by energy-dispersive spectrometry (EDS) are summarized in Table 6. It is seen from Figure 9a that minute fragments of Al-rich IMCs, which have an elemental composition of 23.45 at.% Fe and 76.55 at.% Al, were distributed close to the interfacial layer on the retreating side. An interfacial layer with a thickness of approximately 5 μm, consisting of mixed Fe-rich and Al-rich IMCs, was formed at the joint interface, as shown in Figure 9b. The IMCs formed in the LAFS welds were identified as FeAl, Fe_2_Al_5_, and Fe_4_Al_13_ based on X-ray diffraction (XRD) analysis, as depicted in Figure 10. Comprehensive studies have been conducted on the interfacial formation and growth of intermetallic layers between molten Al and solid Fe. Previous studies have demonstrated that Al-rich phases, such as Fe_2_Al_5_ and FeAl_3_, preferentially form within the temperature range of 700–900 °C. Conversely, IMCs with lower aluminum concentrations, namely FeAl and Fe_3_Al, require significantly higher temperatures exceeding 1000 °C for their formation [23,24,25,26,27]. According to the Al–Fe phase diagram under atmospheric pressure, Fe_3_Al is formed at roughly 552 °C through a first-order reaction of FeAl, whereas FeAl is produced via a peritectic reaction at a much higher temperature of about 1310 °C. Accounting for the low-temperature formation of FeAl and Fe_3_Al requires consideration of the mechanical welding forces applied during FSW. It has been demonstrated that elevated pressure enables interfacial layer formation at lower temperatures for a constant diffusion time [28]. In the FSW process, the combination of vertical and lateral forces raises the localized pressure, driving the intermetallic reactions. Moreover, the severe plastic deformation and high strain rates experienced by the material near the pin significantly accelerate atomic diffusion and IMC evolution. The evolution of the IMC layer is fundamentally governed by the thermal history, mechanical welding forces, and the state of material deformation, all of which are strictly dictated by the selected process parameters [29]. In the LAFSW process, the application of a secondary heat source further modifies these thermomechanical conditions. Hence, these results indicate that laser preheating can enhance the diffusion process and promote the formation of IMCs. Consequently, the elevated temperature induced by the preheating laser significantly affects the formation and growth of the IMCs.

**Figure 11:**
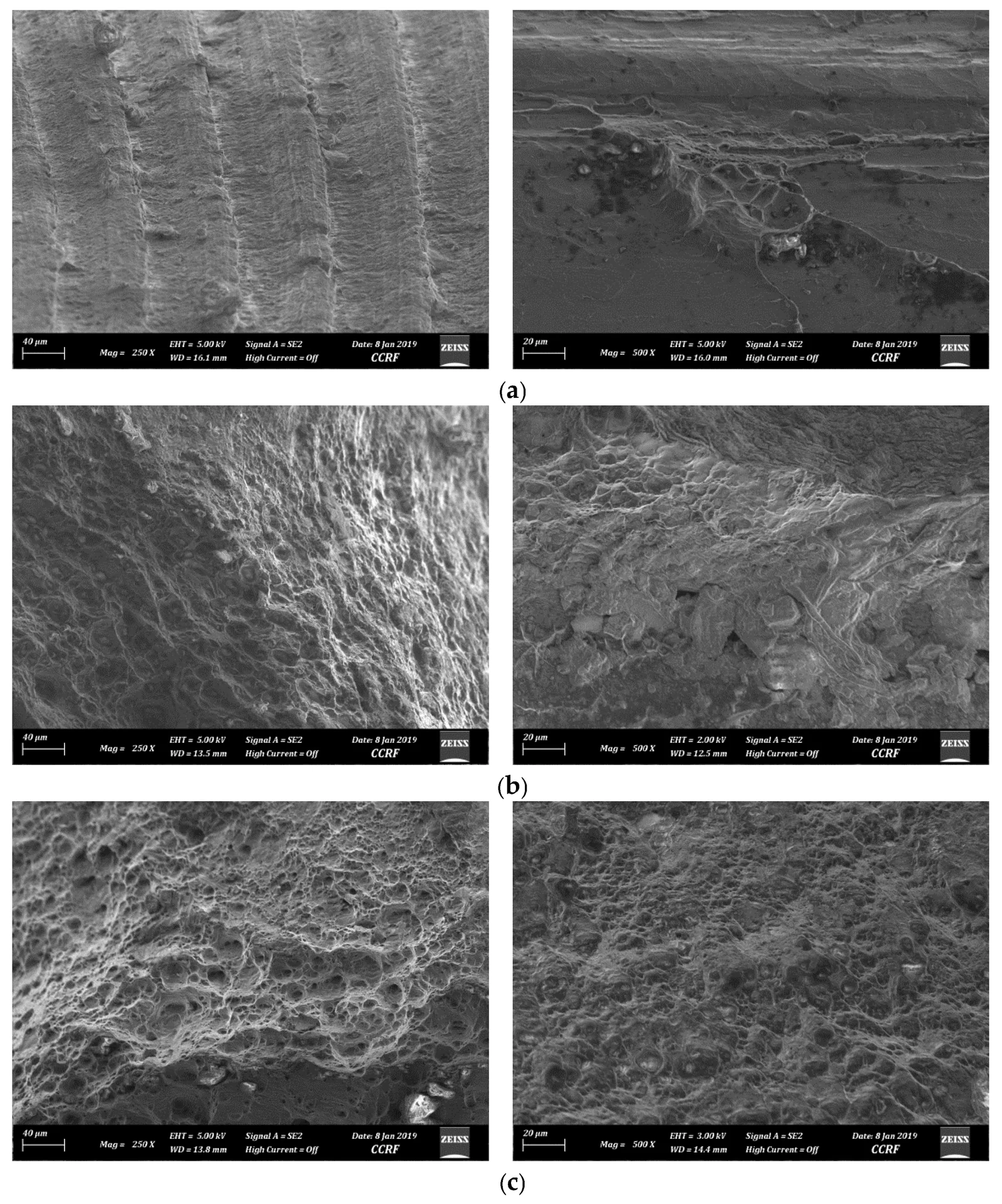
Fracture surface morphologies after tensile test: (**a**) Fracture surface on interface failure mode, (**b**) Fracture surface on interface + NZ failure mode and (**c**) Fracture surface on TMAZ failure mode (left: Al side, and right: steel side) [21].

Figure 11 shows the fracture surface morphologies of the three failure modes observed after the tensile test: interface, interface + NZ, and TMAZ, as previously explained in Figure 5. It is seen from Figure 11a that the fracture surface of the interface failure mode exhibits a cleavage fracture on the Al side, which is significantly influenced by the intermetallic compounds. This failure mode is mainly observed at a lower travel speed of 60 mm/min. When the travel speed is 120 mm/min, a mixed mode characterized by dimples and cleavage features is observed on the fracture surface of the interface + NZ failure mode, as shown in Figure 11b. In the case of the TMAZ failure mode, which corresponds to the highest tensile strength, numerous large and deep dimples with tearing edges associated with micropores are observed on the fracture surfaces of both the Al and steel sides, indicating a typical ductile fracture, as shown in Figure 11c. As previously mentioned, the IMCs scattered in the Al matrix can act as reinforcements. Irregularly sized IMCs were observed on the fracture surface of the Al side. Figure 11c also shows the results obtained from the composition analysis by energy-dispersive spectrometry (EDS) for the intermetallic compounds on the fracture surface, indicating the TMAZ failure mode. These intermetallic compounds have an elemental composition of 29.76 at.% Fe and 70.24 at.% Al. According to the Fe-Al equilibrium phase diagram, this EDS analysis result indicates the presence of the Fe_2_Al_5_ intermetallic compound. Therefore, it can be inferred that these IMCs act as reinforcements in the dissimilar welds, thereby contributing to the enhancement of the tensile strength.

## 4. Conclusions

In the present study, the joining of 2.5 mm thick AA5052-H32 to 1.4 mm thick DP590 steel with dissimilar thicknesses was successfully achieved using LAFSW. Moreover, the crucial effects of process parameters on the mechanical properties and microstructures of the LAFS welds were investigated. The main conclusions can be summarized as follows:

1. The maximum tensile strength of the LAFS welds, produced at a travel speed of 60 mm/min, a rotational speed of 400 rpm, and a laser power of 200 W, was 193 MPa, representing a joint efficiency of approximately 88%. It can be inferred that this increase in tensile strength is attributed to the sufficient material flow facilitated by the elevated temperatures from laser preheating.
2. A localized decrease in hardness was observed in the TMAZ on the retreating side adjacent to the stir zone. This is due to softening caused by a reduction in dislocation density, metallurgical recovery, and the annealing effect during the thermal cycle.
3. An interfacial layer with a thickness of approximately 5 μm was formed in the LAFS welds, consisting of mixed IMCs (FeAl, Fe_2_Al_5_ and Fe_4_Al_13_) as confirmed by XRD analysis. IMC particles scattered in the NZ act as reinforcements in the dissimilar welds, thereby contributing to the enhancement of the tensile strength.
4. The incorporation of a secondary heat source in the LAFSW process modifies the thermomechanical conditions. The elevated temperatures induced by laser preheating significantly enhance atomic diffusion, ultimately driving the formation and growth of the IMCs.

## Figures and Tables

**Table 1:** Chemical compositions and mechanical properties of materials used (as-received) [21].

Chemical Compositions (wt.%)						
Material	C	Mn	Si	P	S	Fe
SPFC590DP	0.0762	1.833	0.249	0.0108	0.0042	Bal.
AA5052-H32	-	0.01	0.09	-	-	0.21
	Mg	Cu	Ti	Cr	Al	
	2.34	0.01	0.011	0.19	97.14	
Mechanical Properties						
Material	Yield strength (MPa)		Tensile strength (MPa)		Elongation (%)	
SPFC590DP	459		623		24	
AA5052-H32	188		225		12	

**Table 2:** Process parameters of LAFSW [21].

Welding Process	Welding Parameters	Values
	Tool rotational speed (rpm)	300, 400, 500
	Travel speed (mm/min)	60, 90, 120
FSW	Plunge depth (mm)	0.9
	Pin-offset (mm)	0.8
	Tilt angle (°)	3 (Fixed)
	Power (W)	200 (Fixed)
Laser	Focal length (mm)	90 (Fixed)
	Beam diameter (mm)	2 (Fixed)
Back plate	STS304L	

**Table 3:** Bead profile of LAFS welds with different travel speeds and rotational speeds [21].

Variables		Travel Speed (mm/min)		
		60	90	120
Rotational speed (rpm)	300	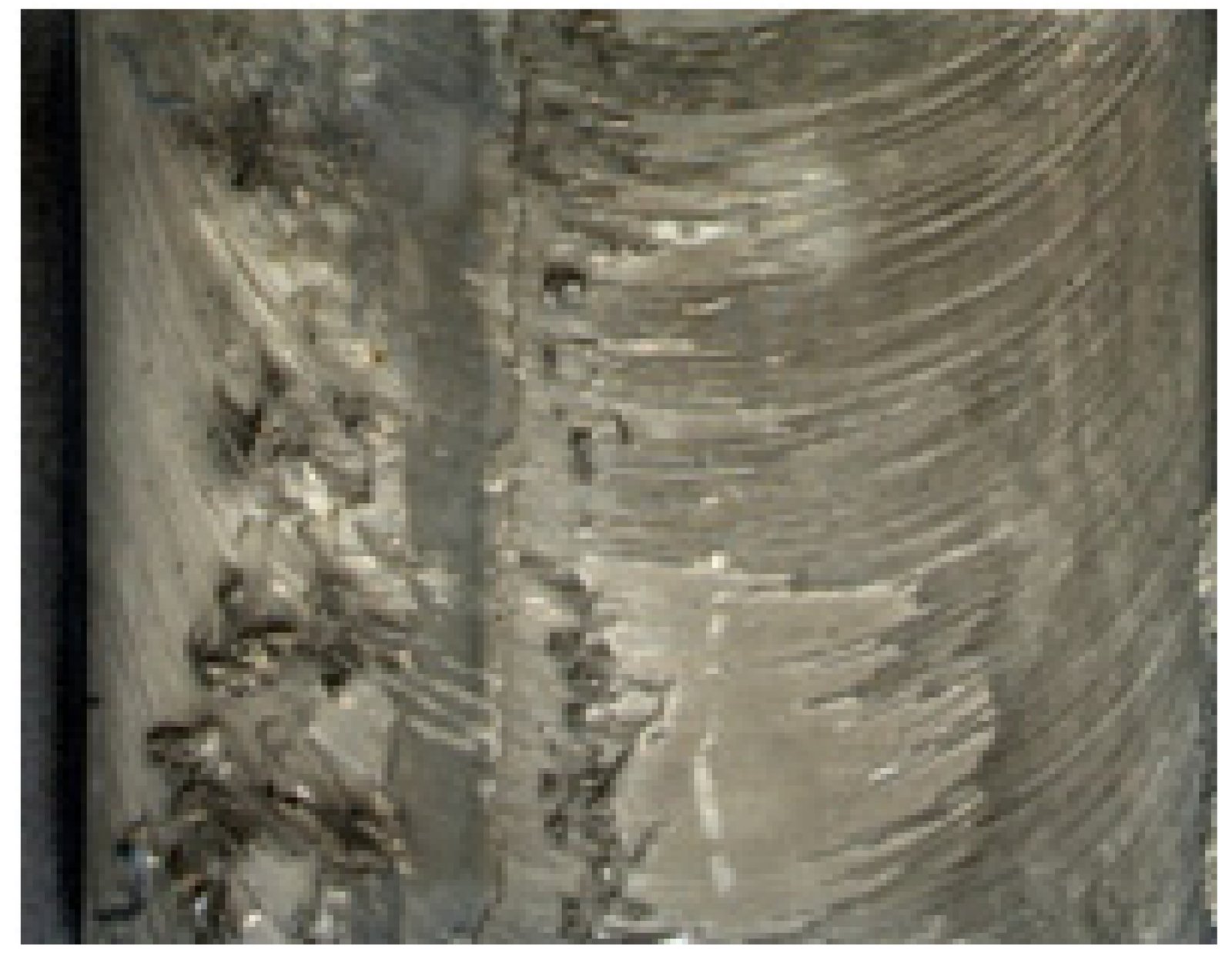	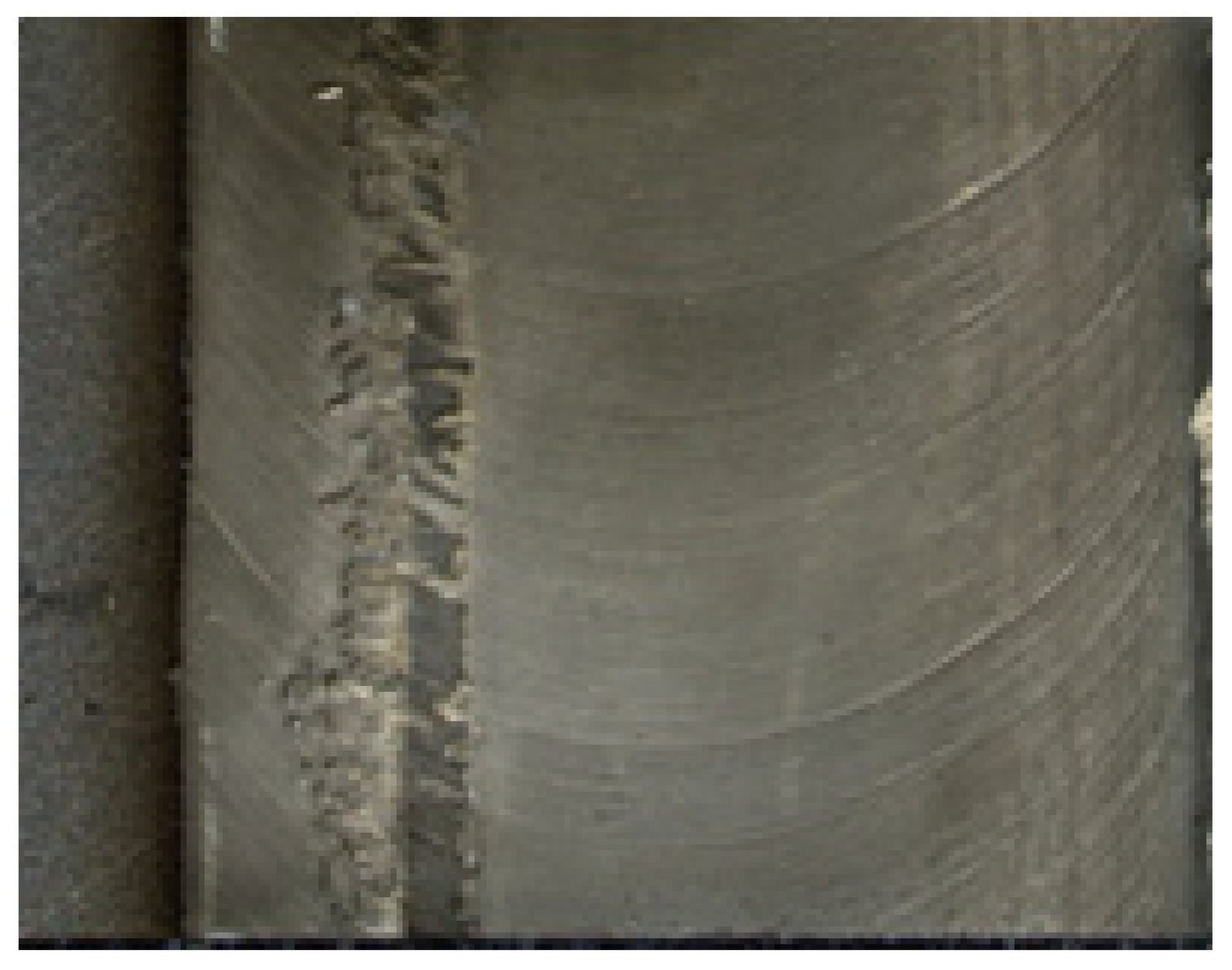	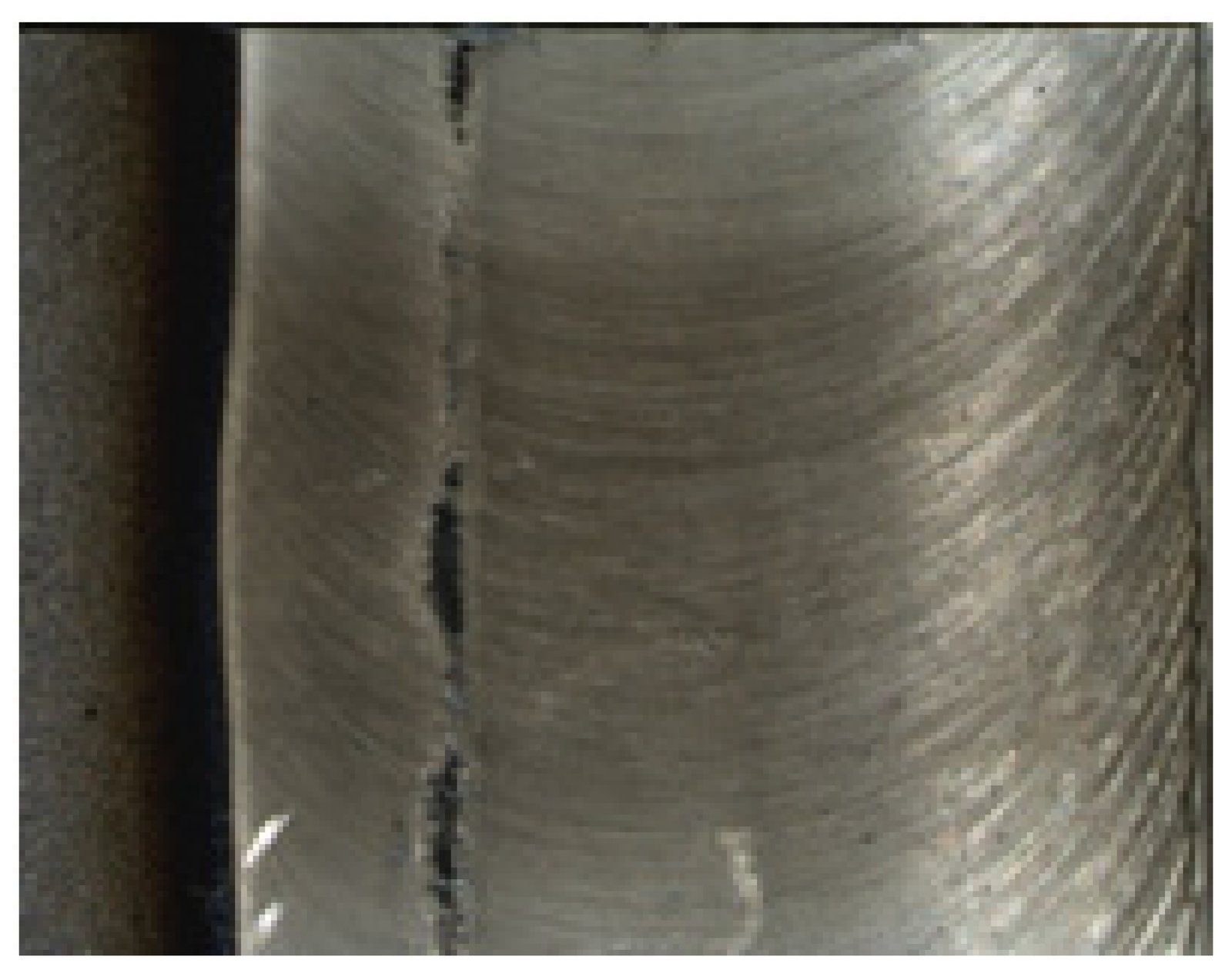
	400	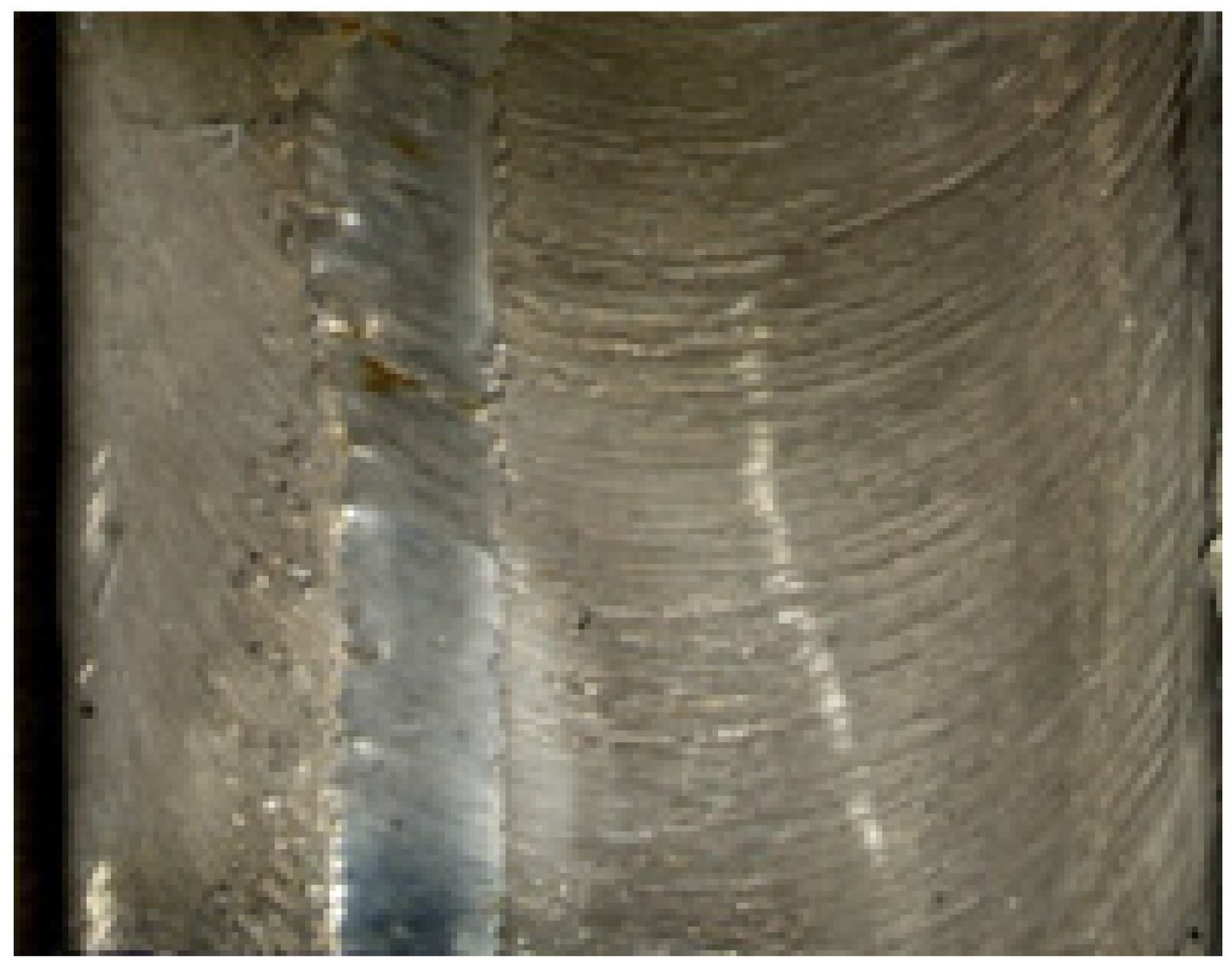	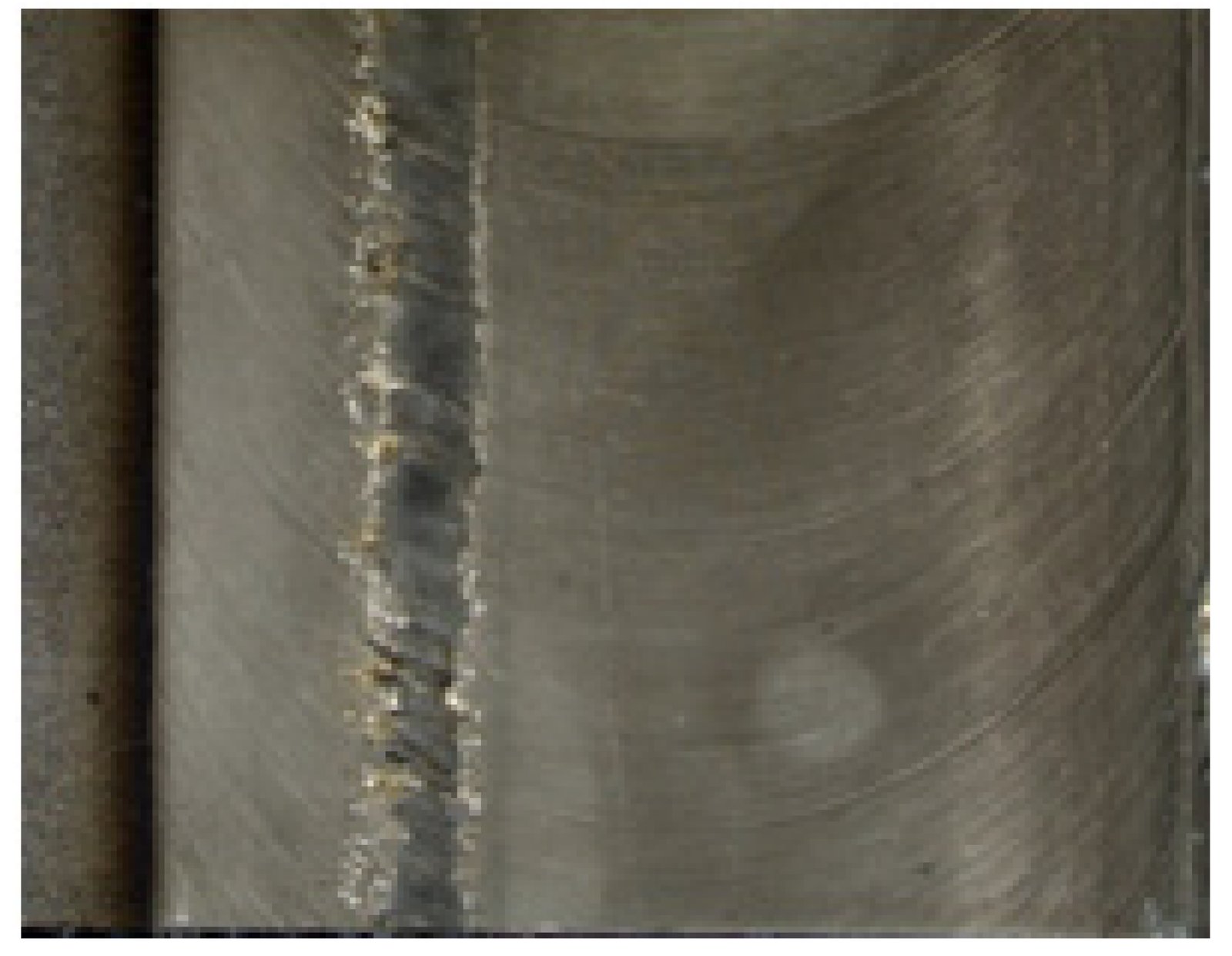	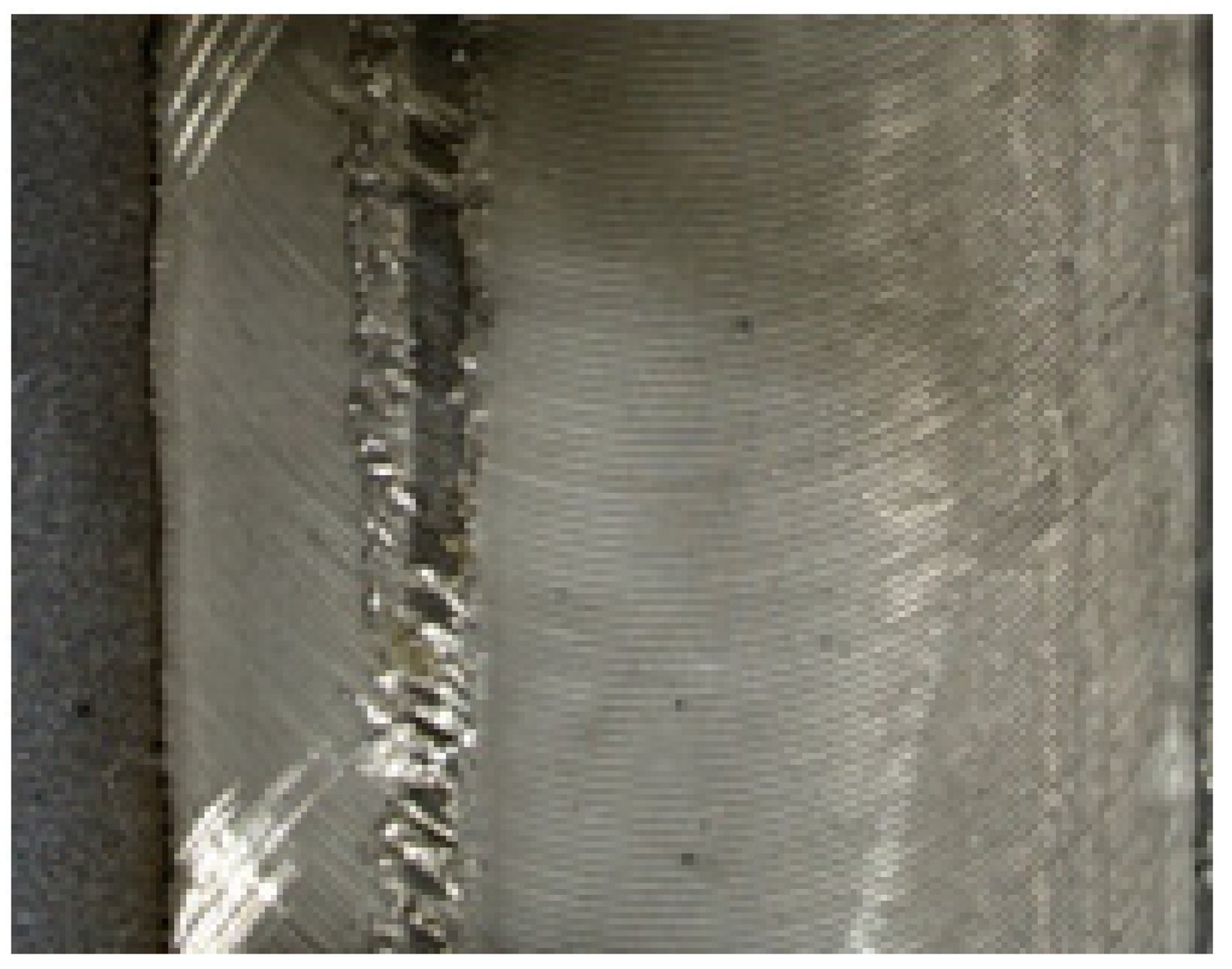
	500	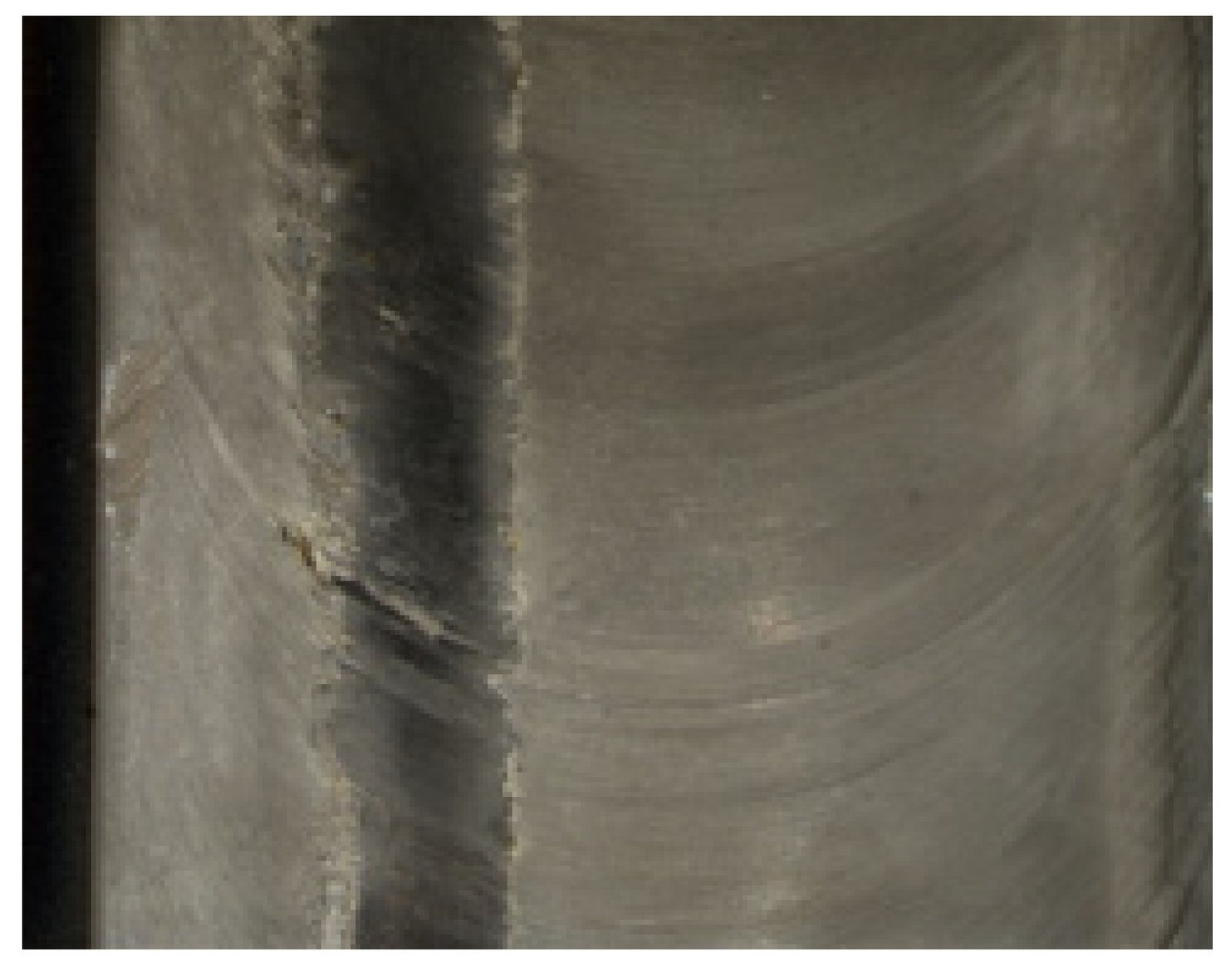	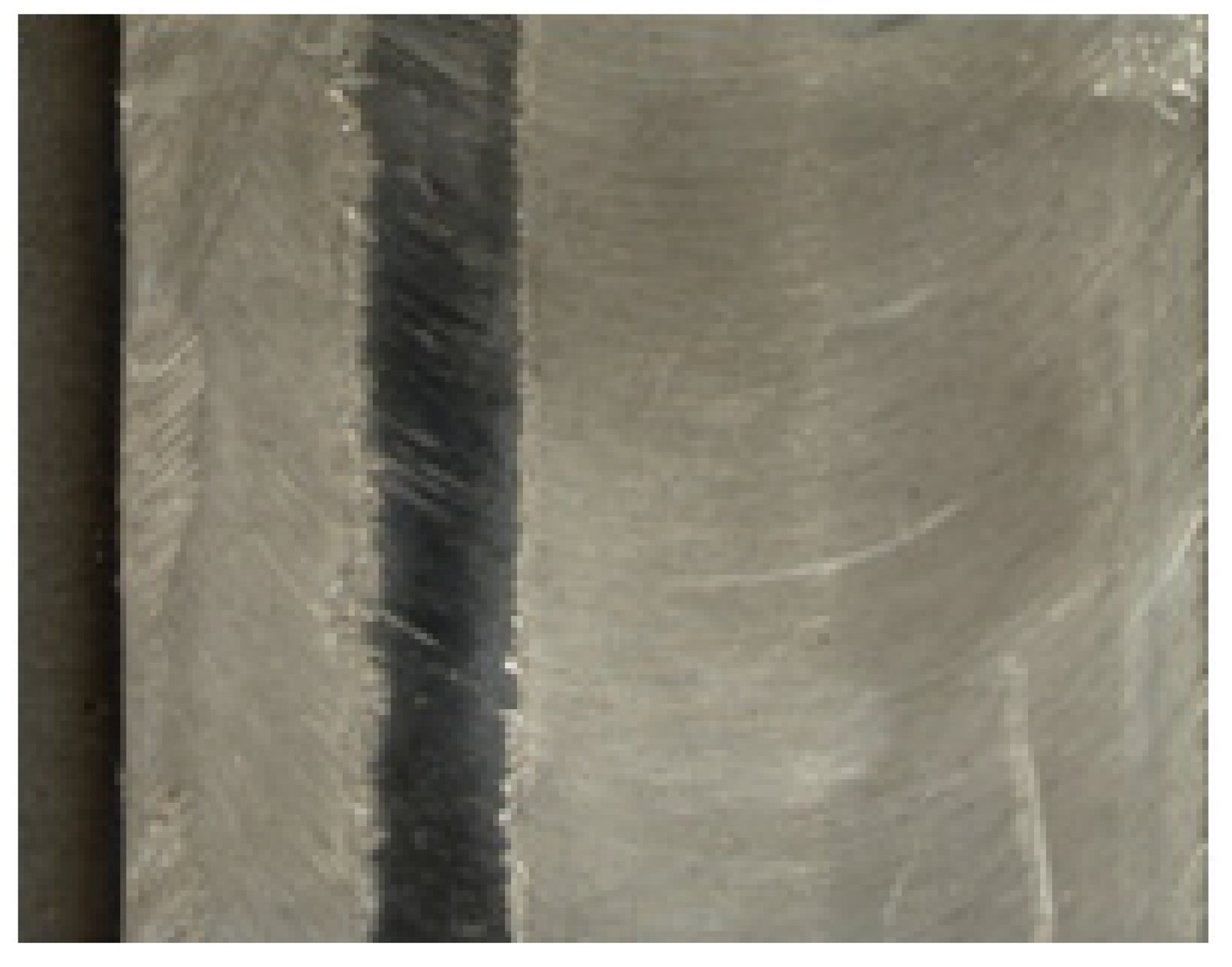	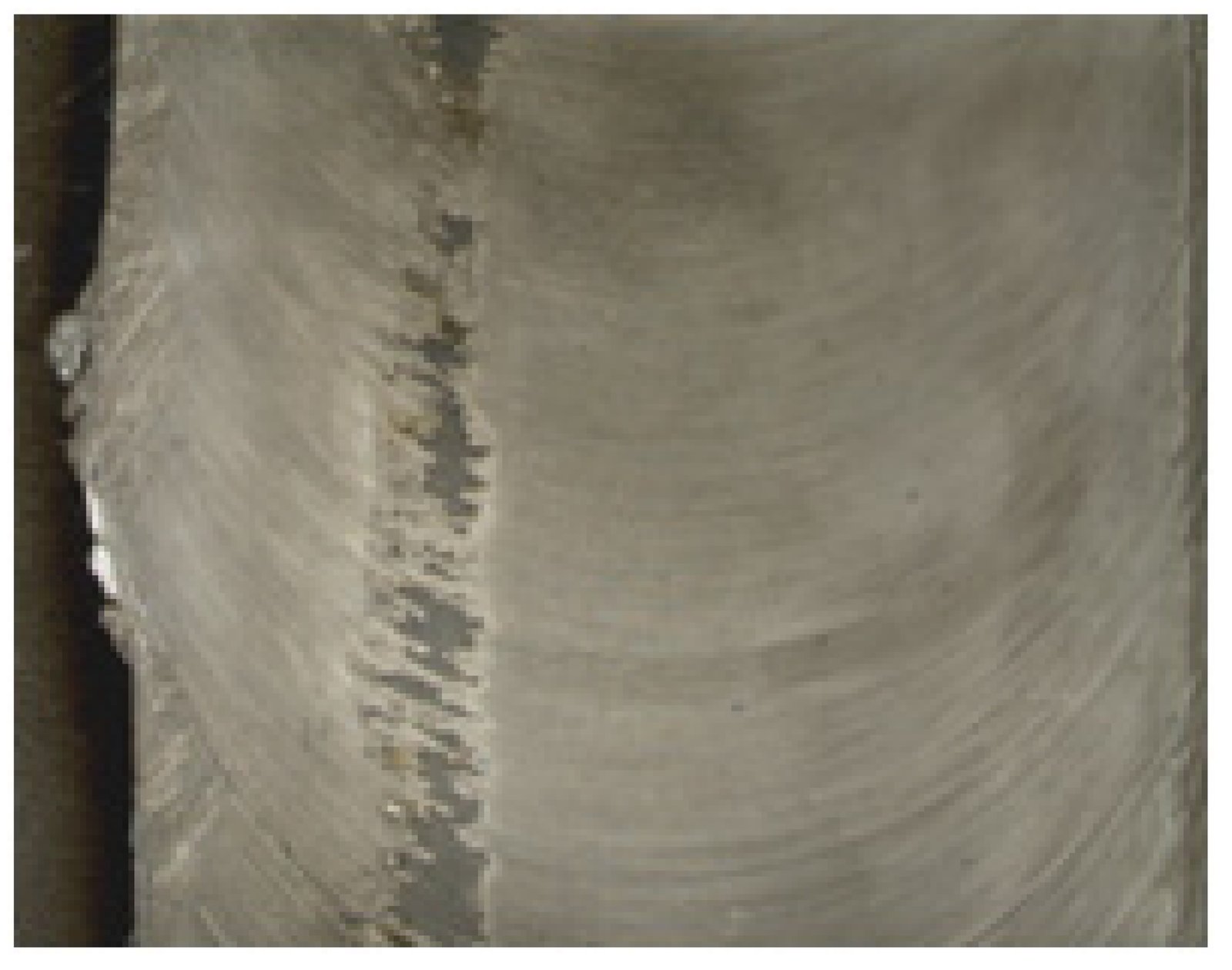

**Table 4:** Cross-sectional view of LAFS welds with different travel speeds and rotational speeds [21].

Variables		Travel Speed (mm/min)		
		60	90	120
Rotational speed (rpm)	300	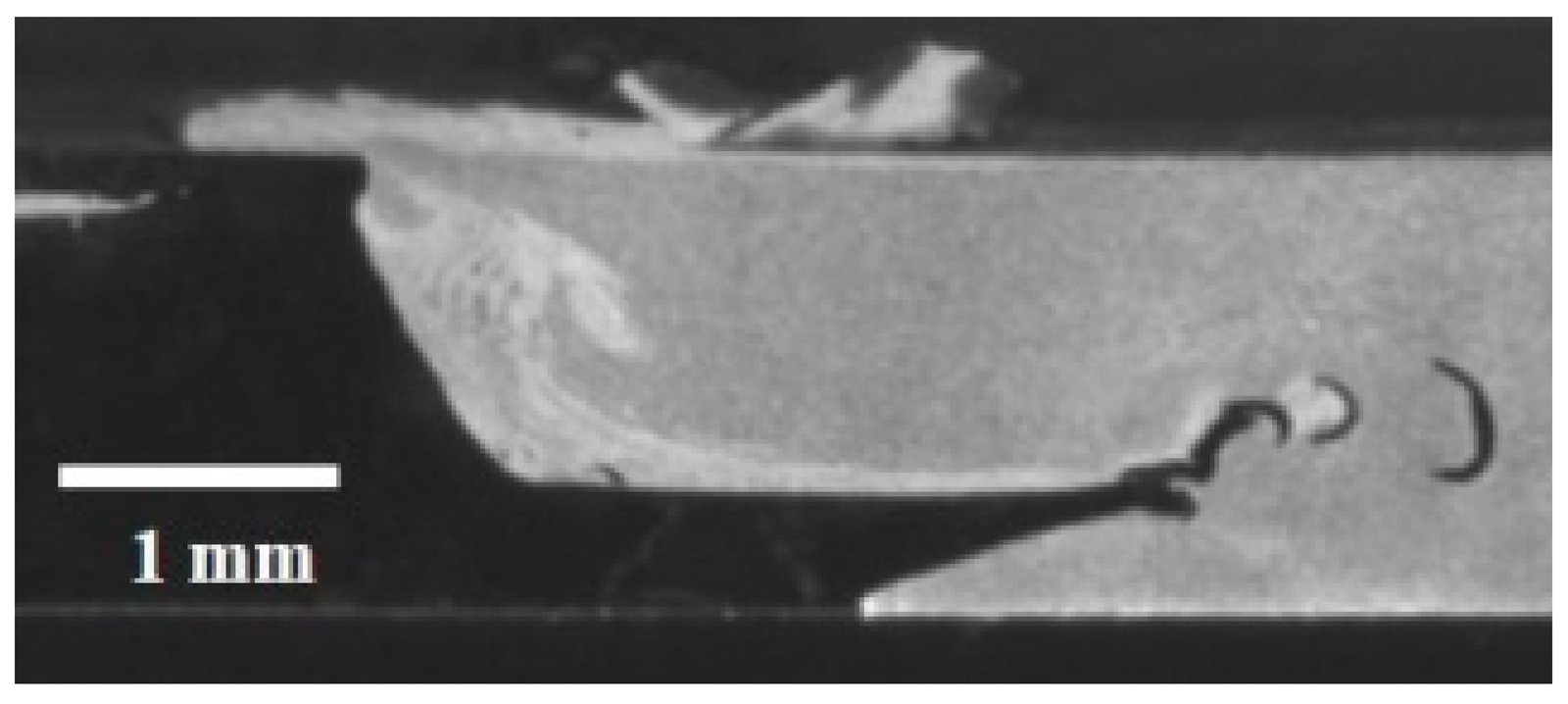	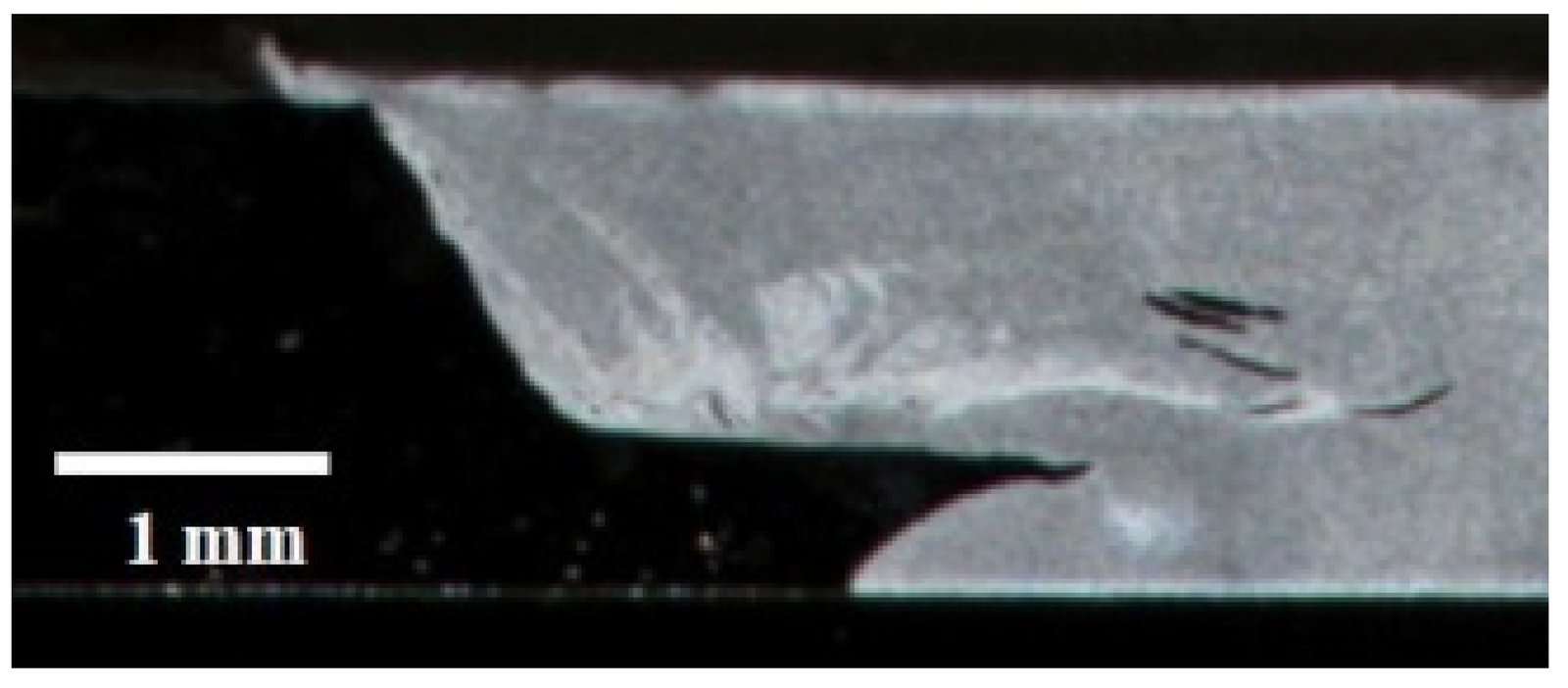	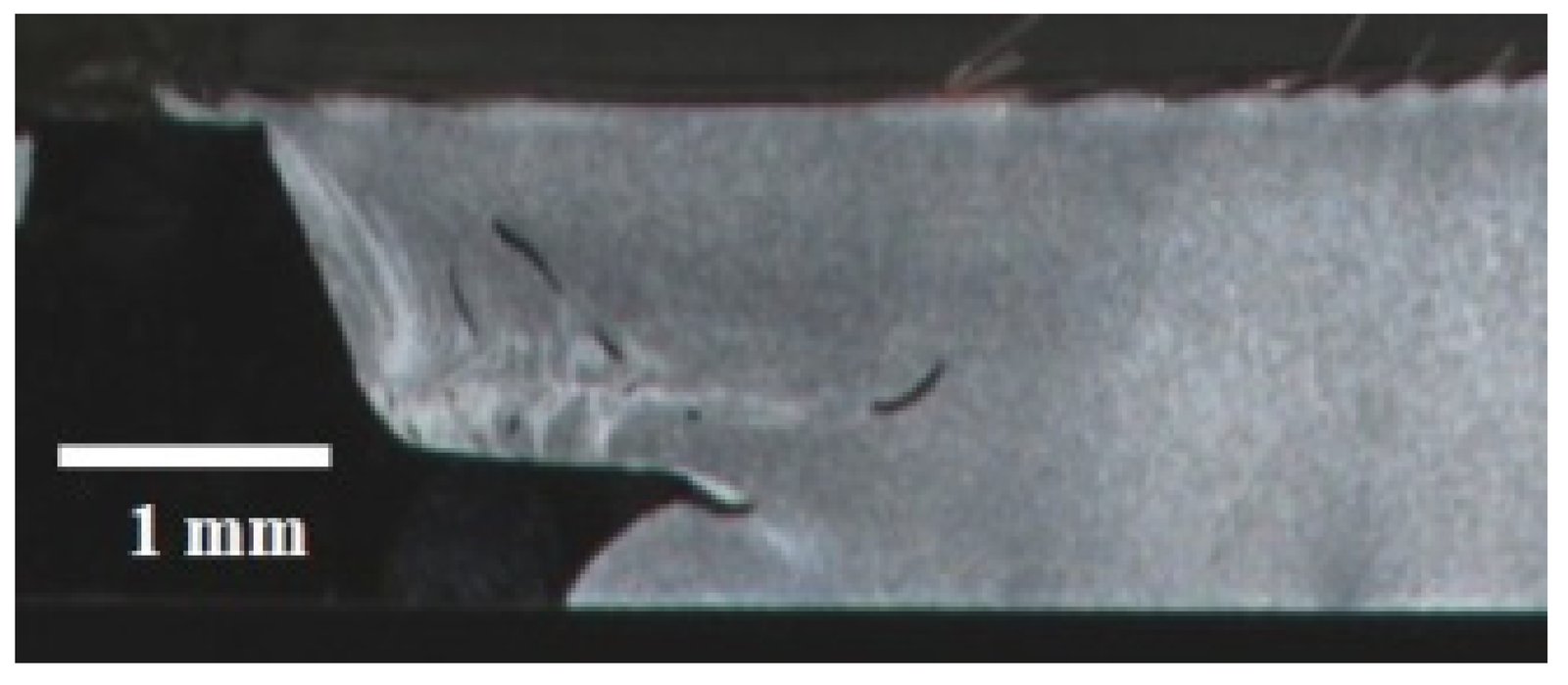
	400	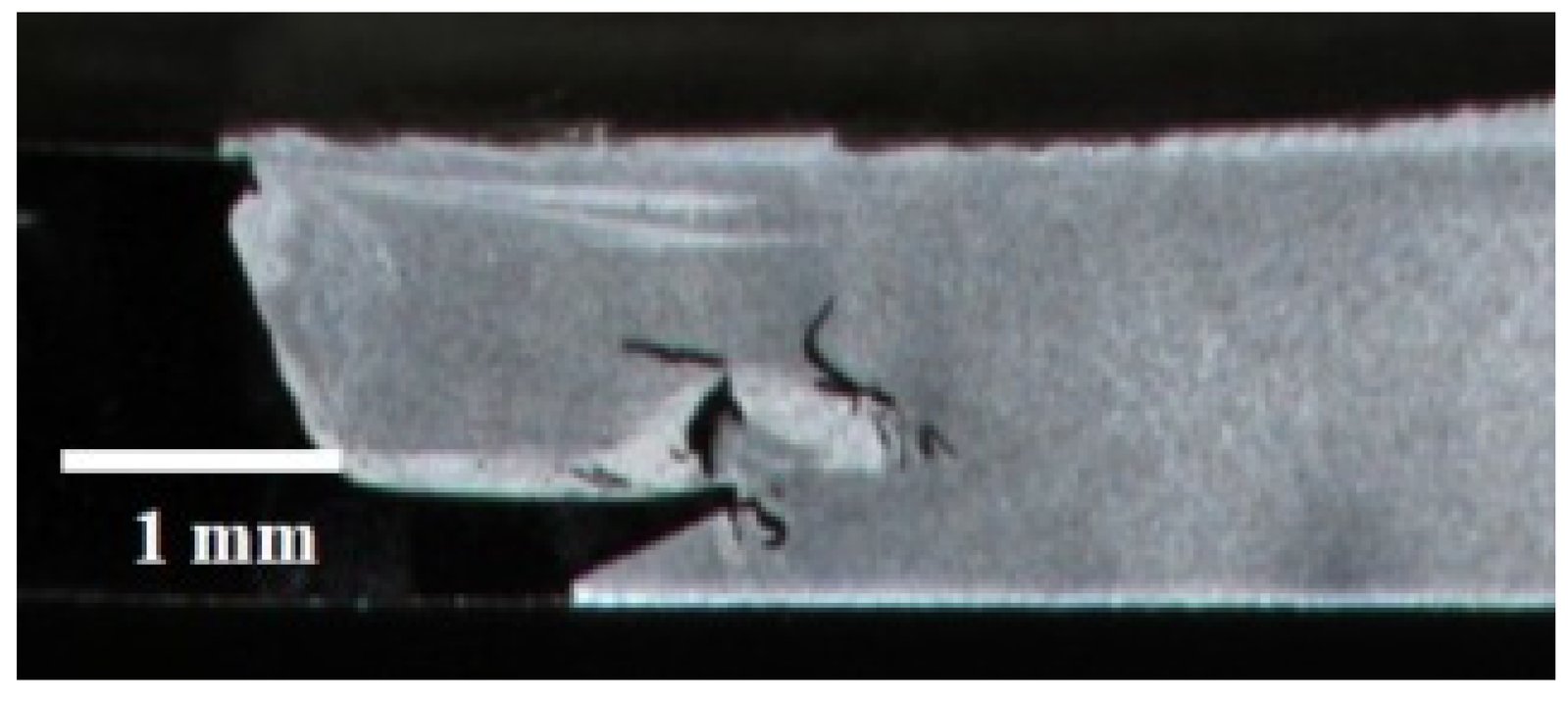	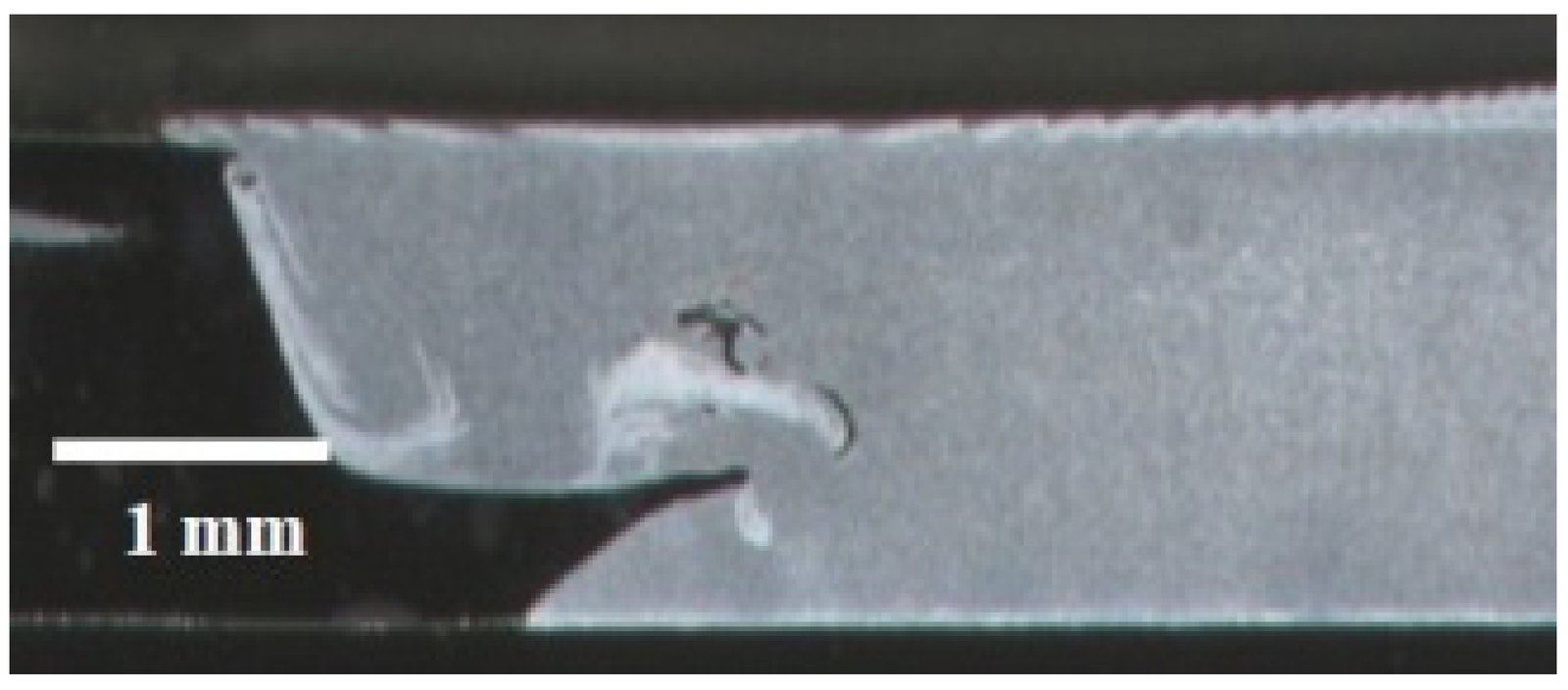	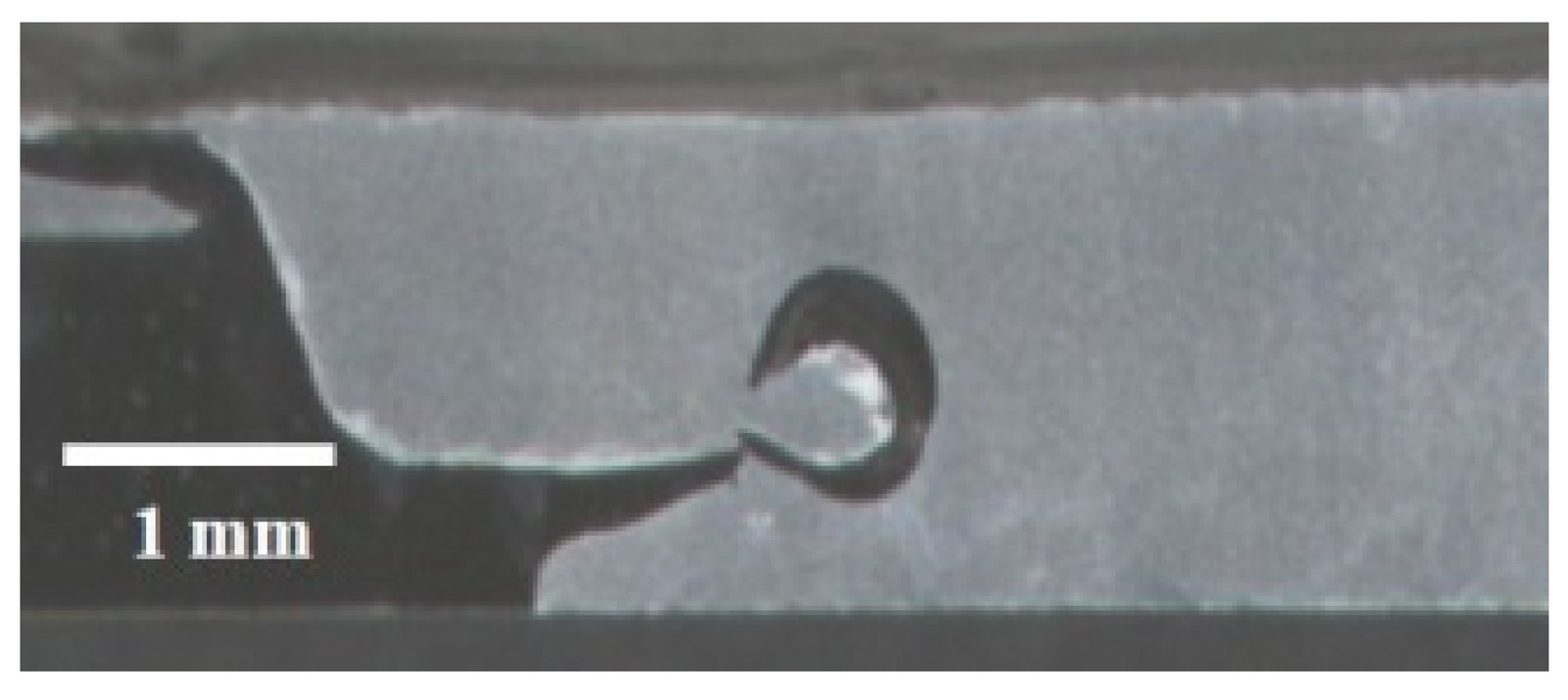
	500	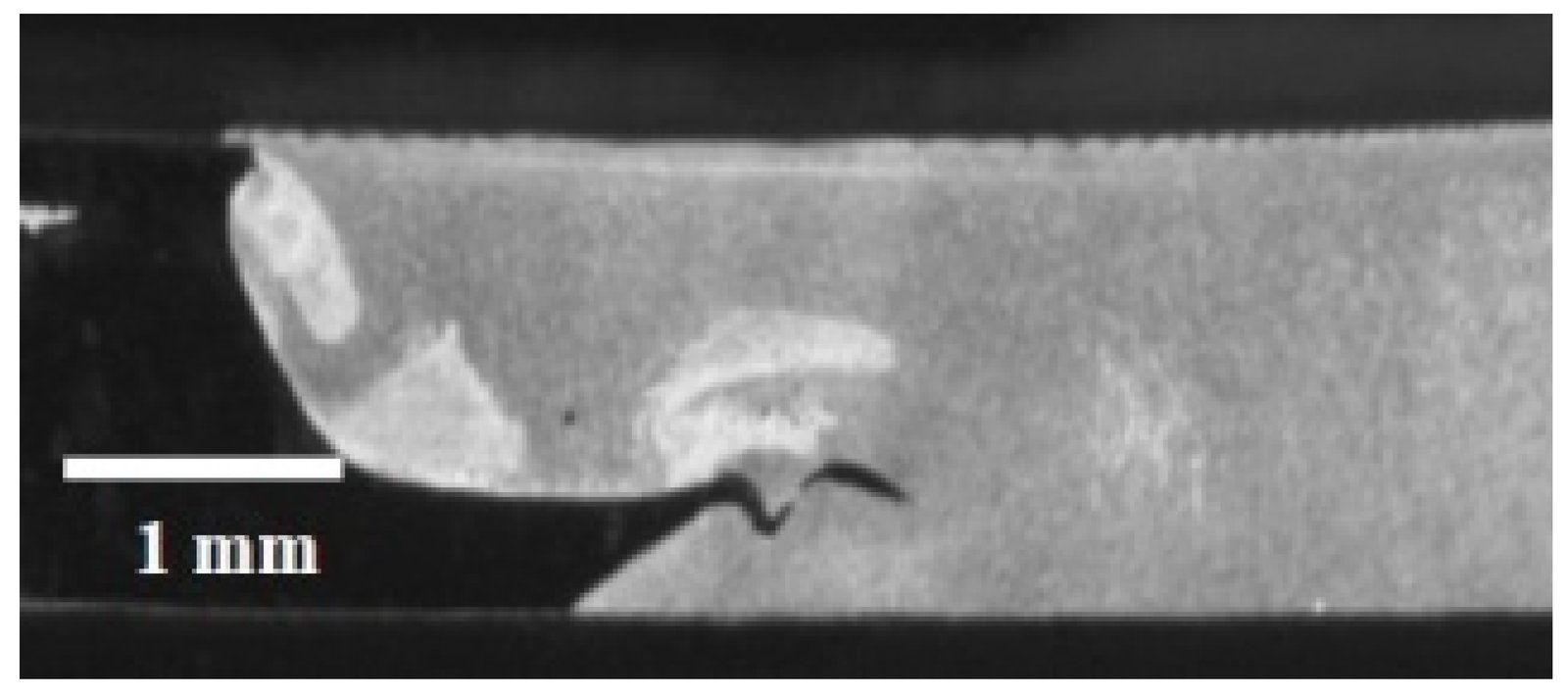	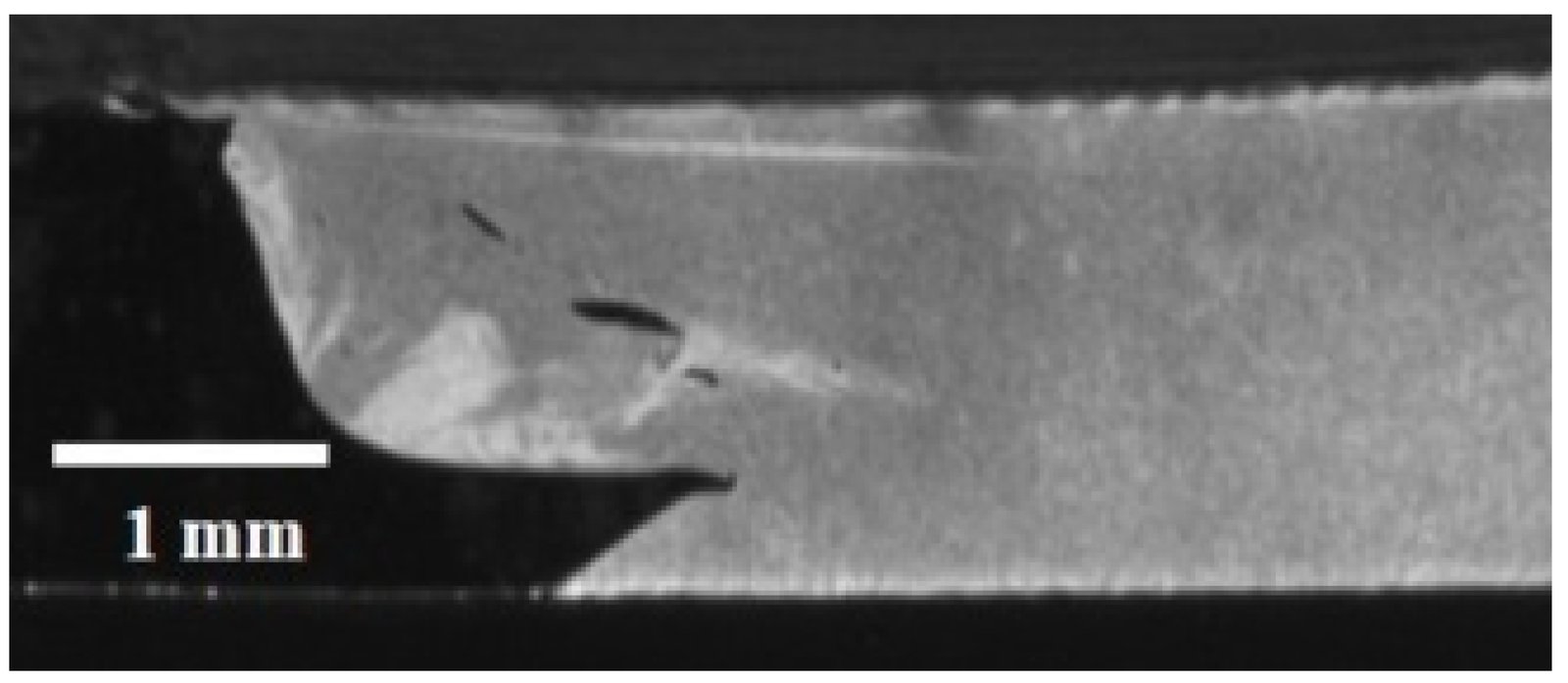	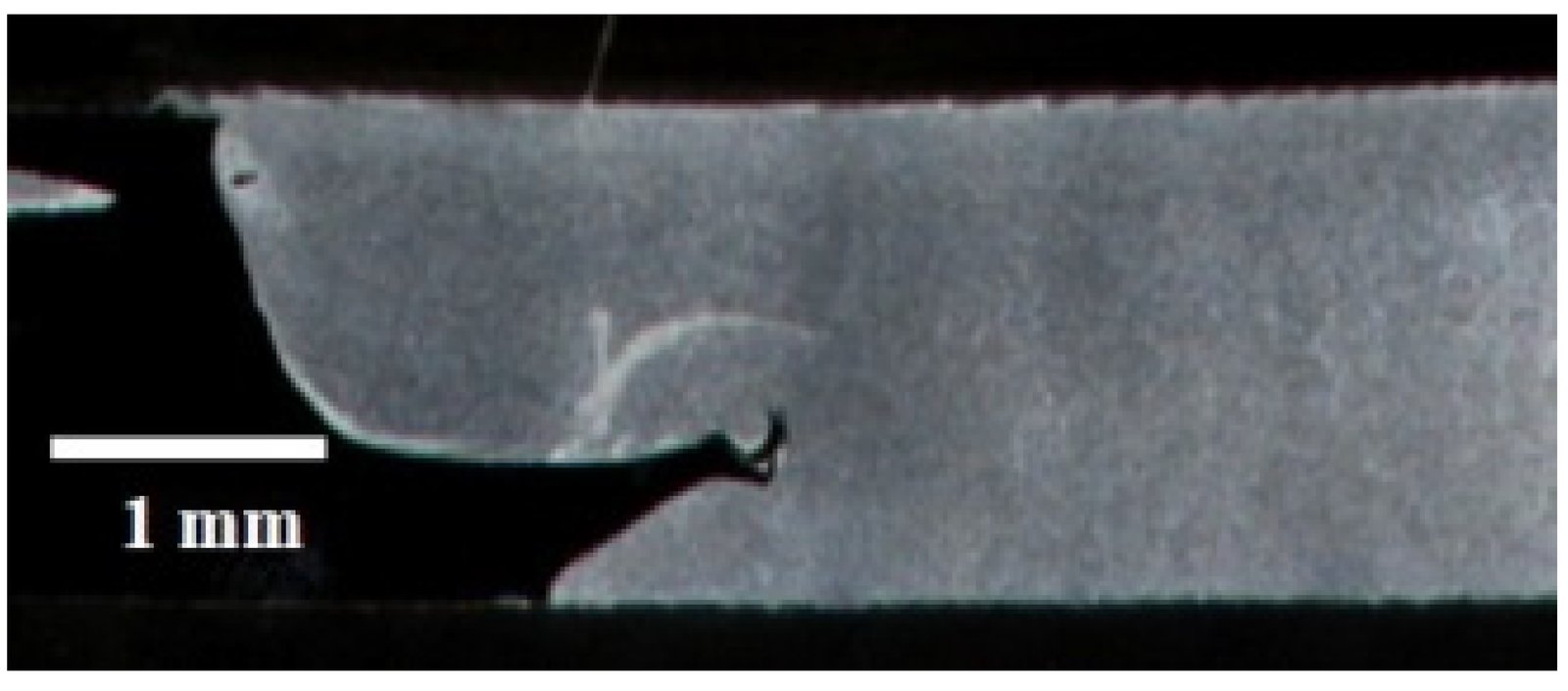

**Table 5:** Fatigue test results of LAFS welds [21].

Smax (MPa)	Δσ (MPa)	(Fmax − Fmin)/2 (kN)	(Fmax + Fmin)/2 (kN)	Nf (×10^3^)
111	100	0.72	0.88	90,867
111	100	0.72	0.88	106,056
111	100	0.72	0.88	128,494
97	87	0.63	0.77	120,590
97	87	0.63	0.77	541,824
97	87	0.63	0.77	542,209
83	75	0.54	0.66	835,492
83	75	0.54	0.66	856,483
83	75	0.54	0.66	981,420
70	63	0.45	0.55	2,000,000
70	63	0.45	0.55	2,000,000
70	63	0.45	0.55	2,000,000

**Table 6:** SEM-EDS results of composition analysis of different areas: (A) at the steel side, (B) Fe-rich intermetallic compounds, (C) Al-rich intermetallic compounds and (D) Intermetallic compound fragments shown in Figure 9b [21].

Area	At.%					
	Fe	Al	C	Mn	Si	P
Point (A)	65.82	-	28.58	1.68	1.21	2.71
Point (B)	72.06	27.94	-	-	-	-
Point (C)	28.05	71.95	-	-	-	-
Point (D)	23.45	76.55				

## Data Availability

The original contributions presented in this study are included in the article. Further inquiries can be directed to the corresponding authors.

## References

[1] Sierra G., Peyre F., Deschaux Beaume F., Stuart D., Fras G. (2008). Steel to Aluminium Braze Welding by Laser Process with Al–12Si Filler Wire. Sci. Technol. Weld. Join..

[2] Nasiri A., Li L., Kim S.H., Zhou Y., Weckman D.C., Nguyen T.C. (2011). Microstructure and Properties of Laser Brazed Magnesium to Coated Steel. Weld. J..

[3] Pouranvari M. (2017). Critical Assessment 27: Dissimilar Resistance Spot Welding of Aluminium/Steel: Challenges and Opportunities. Mater. Sci. Technol..

[4] Karfoul M.K., Tatlock G.J., Murray R.T. (2007). The Behavior of Iron and Aluminium during the Diffusion Welding of Carbon Steel to Aluminium. J. Mater. Sci..

[5] Yu H., Xu Z., Fan Z., Zhao Z., Li C. (2013). Mechanical Property and Microstructure of Aluminum Alloy-Steel Tubes Joint by Magnetic Pulse Welding. Mater. Sci. Eng. A.

[6] Prangnell P., Haddadi F., Chen Y.C. (2011). Ultrasonic Spot Welding of Aluminium to Steel for Automotive Applications—Microstructure and Optimisation. Mater. Sci. Technol..

[7] Ratanathavorn W., Melander A., Magnusson H. (2016). Intermetallic Compounds in Friction Stir Lap Joints between AA5754/Galvanized Ultra-High Strength Steel. Sci. Technol. Weld. Join..

[8] Bang H.S., Bang H.S., Jeon G.H., Oh I.H., Ro C.S. (2012). Gas Tungsten Arc Welding Assisted Hybrid Friction Stir Welding of Dissimilar Materials Al6061-T6 Aluminum Alloy and STS304 Stainless Steel. Mater. Des..

[9] Song J.L., Lin S.B., Yang C.L., Fan C.L., Ma G.C. (2010). Analysis of Intermetallic Layer in Dissimilar TIG Welding-Brazing Butt Joint of Aluminium Alloy to Stainless Steel. Sci. Technol. Weld. Join..

[10] Mei S.W., Gao M., Yan J., Zhang C., Li G., Zeng X.Y. (2013). Interface Properties and Thermodynamic Analysis of Laser-Arc Hybrid Welded Al/Steel Joint. Sci. Technol. Weld. Join..

[11] Chen N., Wang H.P., Carlson B.E., Sigler D.R., Wang M. (2018). Fracture Mechanisms of Al/steel Resistance Spot Welds in Coach Peel and Cross Tension Testing. J. Mater. Process. Technol..

[12] Rakhi K., Kang S., Shin J. (2023). Hot-Cracking Mechanism of Laser Welding of Aluminum Alloy 6061 in Lap Joint Configuration. Materials.

[13] Nagatsuka K., Xiao B., Wu L., Nakata K., Saeki S., Kitamoto Y., Iwamoto Y. (2018). Resistance Spot Welding of Metal/Carbon-Fibre-Reinforced Plastics and Applying Silane Coupling Treatment. Sci. Technol. Weld. Join..

[14] Kim K.H., Bang H.S., Ro C.S., Bang H.S. (2017). Influence of Preheating Source on Mechanical Properties and Welding Residual Stress Characteristics in Ultra Thin Ferritic Stainless Steel Hybrid Friction Stir Welded Joints. Int. J. Precis. Eng. Manuf.-Green Technol..

[15] Bang H.S., Bang H.S., Song H.J., Joo S.M. (2013). Joint Properties of Dissimilar Al6061-T6 Aluminum Alloy/Ti-6%Al-4%V Titanium Alloy by Gas Tungsten Arc Welding Assisted Hybrid Friction Stir Welding. Mater. Des..

[16] Bang H.S., Hong S.M., Das A., Bang H.S. (2020). A Prediction of Fe-Al IMC Layer Thickness in TIG-Assisted Hybrid Friction Stir Welded Al/Steel Dissimilar Joints by Numerical Analysis. Int. J. Adv. Manuf. Technol..

[17] Bang H.S., Hong S.M., Das A., Bang H.S. (2021). Study on the Weldability and Mechanical Characteristics of Dissimilar Materials (Al5052-DP590) by TIG Assisted Hybrid Friction Stir Welding. Met. Mater. Int..

[18] Merklein M., Giera A. (2008). Laser Assisted Friction Stir Welding of Drawable Steel-Aluminum Tailored Hybrids. Int. J. Mater. Form..

[19] Bang H.S., Bang H.S., Kim H.S., Kim J.H., Oh I.H., Ro C.S. (2010). A Study on the Weldability and Mechanical Characteristics of Dissimilar Materials Butt Joints by Laser Assisted Friction Stir Welding. J. Weld. Join..

[20] Bang H.S., Bang H.S., Kim K.H. (2021). Effects of Process Parameters on Friction Stir Weldability in Dissimilar Joints of AA5052 and Advanced High Strength Steel. J. Weld. Join..

[21] Kim K.H. (2019). The Weldability of Aluminium Alloy and Advanced High Strength Steel with Different Thicknesses by Hybrid Friction Stir Welding. Doctoral Thesis.

[22] You H.J., Kang M.J., Yi S., Hyun S.K., Kim C.H. (2021). Comprehensive Analysis of the Microstructure and Mechanical Properties of Friction-Stir-Welded Low-Carbon High-Strength Steels with Tensile Strengths Ranging from 590 MPa to 1.5 GPa. Appl. Sci..

[23] Bouché K., Barbier F., Coulet A. (1998). Intermetallic Compound Layer Growth between Solid Iron and Molten Aluminium. Mater. Sci. Eng. A.

[24] Kobayashi S., Yakou T. (2002). Control of Intermetallic Compound Layers at Interface between Steel and Aluminum by Diffusion-Treatment. Mater. Sci. Eng. A.

[25] Bouayad A., Gerometta C.H., Belkebir A., Ambari A. (2003). Kinetic Interactions between Solid Iron and Molten Aluminium. Mater. Sci. Eng. A.

[26] Yeremenko V.N., Natanzon Y.V., Dybkov V.I. (1981). The Effect of Dissolution on the Growth of the Fe_2_Al_5_ Interlayer in the Solid Iron-Liquid Aluminium System. J. Mater. Sci..

[27] Shahverdi H.R., Ghomashchi M.R., Shabestari S., Hejazi J. (2002). Microstructural Analysis of Interfacial Reaction between Molten Aluminium and Solid Iron. J. Mater. Process. Technol..

[28] Rathod M.J., Kutsuna M. (2004). Joining of Aluminum Alloy 5052 and Low-Carbon Steel by Laser Roll Welding. Weld. J..

[29] Liu X., Lan S., Ni J. (2014). Analysis of Process Parameters Effects on Friction Stir Welding of Dissimilar Aluminum Alloy to Advanced High Strength Steel. Mater. Des..

